# Enzymatic property and stabilization mechanism of LysBT1, a novel polyextremotolerant endolysin with a C-terminal S-layer homology domain

**DOI:** 10.1128/aem.00867-25

**Published:** 2025-06-13

**Authors:** Yu Li, Ke Luo, Chaofeng Jiang, Yihao Zhang, Yong Yang, Yitong Yao, Huai Li, Fei Gan, Xiao-Feng Tang, Bing Tang

**Affiliations:** 1 State Key Laboratory of Virology, College of Life Sciences, Wuhan University 98436 , Wuhan, China; 2 Hubei Key Laboratory of Cell Homeostasis, College of Life Sciences, Wuhan University 98436 , Wuhan, China; 3 Cooperative Innovation Center of Industrial Fermentation (Ministry of Education & Hubei Province), Wuhan, China; University of Nebraska-Lincoln, Lincoln, Nebraska, USA

**Keywords:** endolysin, N-acetylmuramoyl-L-alanine amidase, S-layer homology domain, prophage, thermophile, antimicrobials

## Abstract

Phage-encoded endolysins are getting increasing attention because of their potential to serve as alternative antimicrobials to combat antibiotic-resistant bacteria. Here, we report a novel endolysin LysBT1, which is encoded by a prophage of thermophilic *Brevibacillus thermoruber* WF146 and comprises an N-acetylmuramoyl-L-alanine amidase domain and an S-layer homology (SLH) domain not found in known endolysins. LysBT1 is not only extremely thermostable, retaining more than 60% activity after 1 h incubation at 95°C, but also highly stable over a wide pH range of 4.0–11.0. Moreover, the thermostability of LysBT1 could be enhanced by EDTA or reducing agents. Although none of the seven cysteine residues of LysBT1 participate in disulfide bond formation, six of them, including the catalytic Zn^2+^-coordinating Cys156, are involved in stabilizing the enzyme at elevated temperatures. The SLH domain contributes to the thermostability of LysBT1 and mediates cell surface binding of the enzyme to facilitate enzymatic lysis of strain WF146 cells via increasing local enzyme concentration around the substrate. LysBT1 is capable of trimerization, where the SLH domains are predicted to form a three-prong spindle-like trimer similar to that in S-layer proteins. The SLH domain of LysBT1 could bind to cell surfaces of both Gram-positive and Gram-negative bacteria. LysBT1 can lyse not only Gram-positive strain WF146, *Geobacillus stearothermophilus*, and *Bacillus subtilis* but also Gram-negative *Escherichia coli* and *Acinetobacter baumannii* with the aid of EDTA or citric acid. EDTA also facilitates LysBT1 to lyse *Bacillus cereus*, probably because EDTA-induced disorganization of the S-layer allows LysBT1 to access and hydrolyze the peptidoglycan.

**IMPORTANCE:**

The emergence of antibiotic-resistant bacteria has led to an urgent requirement to develop novel antimicrobials, and endolysins are regarded as ideal alternatives to antibiotics. The thermostability of endolysins plays an important role in the feasibility of enzymatic bacteriolysis. However, reports on thermostable endolysins are limited, and little is known about their stabilization mechanisms. Our results demonstrate that the thermophile-derived prophage endolysin LysBT1 is highly thermostable and functional under polyextreme (multiple forms of stress) conditions, enabling the enzyme to lyse both Gram-positive and Gram-negative bacteria in synergy with outer membrane permeabilizer. Moreover, we found that the unique S-layer homology domain of LysBT1 contributes to the stability, activity, oligomerization, and cell-wall binding ability of the enzyme. This study not only characterizes a novel endolysin but also provides new clues about the stabilization mechanisms of endolysins.

## INTRODUCTION

The overuse of antibiotics has favored the emergence of antibiotic-resistant bacteria, raising global health threats. There is an urgent need to develop novel antimicrobials to treat bacterial infections. Recently, there has been a renewed interest in exploiting the potential of phages and their lytic enzymes such as endolysins as antimicrobials to combat antibiotic-resistant bacteria (1, 2). Nevertheless, bacteria can also readily evolve phage resistance by multiple tactics such as phage receptor modification, superinfection exclusion, restriction-modification system, and CRISPR-Cas system (3). Moreover, phages can transfer undesirable genes (e.g., antibiotic-resistance genes) between host bacteria via specialized or generalized transduction (4). By contrast, the occurrence of bacterial resistance to endolysins is rare, probably because the target of endolysins is peptidoglycan that is essential for bacterial viability, and thus, adaptive mutations leading to endolysin resistance would be too harmful to bacterial cells (1, 2). Compared with phages, endolysins also have the advantage of a relatively broad spectrum of antimicrobial activity, versatility in synergism with different antimicrobial agents, convenient preparation and storage, etc. (2). Endolysins can be applied externally to destroy the peptidoglycan of Gram-positive bacteria, and some endolysins such as LysK from staphylococcal phage K show lytic activity against methicillin-resistant *Staphylococcus aureus* (MRSA) (5). Meanwhile, endolysins can act on Gram-negative bacteria when used in combination with outer membrane (OM) permeabilizers (1, 2).

At the late stage of the phage reproductive cycle, endolysins pass through the holes formed by phage-encoded holins in the cytoplasmic membrane to degrade peptidoglycan, leading to the release of phage particles and cell lysis (canonical lysis). Some endolysins possess N-terminal signal peptide or signal-anchor-release (SAR) domain and transport across the cytoplasmic membrane via the Sec pathway to degrade peptidoglycan (non-canonical lysis) (6). Endolysins targeting Gram-positive bacteria are generally modular proteins consisting of the N-terminal enzymatically active domain(s) (EAD) and the C-terminal cell wall-binding domain(s) (CBD), while those targeting Gram-negative bacteria usually comprise only a single EAD (7). According to their hydrolytic cleavage sites within the peptidoglycan, endolysins can be subdivided into N-acetylglucosaminidases, N-acetylmuramidases, transglycosylases, N-acetylmuramoyl-L-alanine amidases (NALAAs), endopeptidases, and cysteine histidine-dependent amidohydrolase/peptidases (CHAPs) (7, 8). Characterized NALAA endolysins belong to either the Amidase_2 family, which is exemplified by T7 lysozyme from the *Escherichia coli* phage T7 (9), or the Amidase_3 family (8). Based on their structure features, the CBDs of endolysins have been grouped broadly into four main classes: choline-binding modules, SH3b domains, three-helix bundles, and α/β multimers (7). Nevertheless, some CBDs such as that of BlyA from the *Bacillus subtilis* 168 prophage SPβ (10) do not show structural similarity to the four classes. In general, the CBDs bind to ligand molecules within cell wall and contribute to the specificity of endolysins for their bacterial hosts (7). CBDs can also modulate the lytic activity of endolysins. For instance, the deletion of the CBD of XlyA from the *B. subtilis* 168 prophage PBSX leads to a loss of activity of the endolysin (11). By contrast, the CBD of the *Bacillus anthracis* prophage endolysin PlyL inhibits the activity of its cognate EAD through intramolecular interactions that are relieved upon binding of the CBD to the cell wall (12).

Surface-layer homology (SLH) domains are often present in single, duplicate, or triplicate copies at the termini of S-layer proteins and some extracellular enzymes or OM proteins of bacteria (13). The three tandem SLH domains of the S-layer proteins of *B. anthracis* (Sap) and *Paenibacillus alvei* (SpaA) fold into a three-prong spindle-like pseudotrimer (14, 15), and the single SLH domain of the OM protein SlpA can form a homotrimer structurally similar to the pseudotrimer of Sap or SpaA (16). SLH domains have a conserved sequence of ~55 amino acid residues and are able to non-covalently bind to secondary cell wall polysaccharide (SCWP) (14–17). However, to date, no SLH domain-containing endolysin has been reported.

Given the proteinaceous nature of endolysins, thermostability directly correlates with their preparation efficiency, storage, shelf life, catalytic behavior, and compatibility with other additives (e.g., OM permeabilizing agents) (1, 18). Moreover, thermostable endolysins can be exploited for a wider range of applications, especially because mesophilic endolysins may face stability problems, such as food and feed production processes involving heat treatment (19). Many efforts have been made to stabilize endolysins by protein engineering, immobilization, and formulation methods (1, 18). Meanwhile, thermophilic phage-derived NALAAs belonging to the Amidase_2 family (20–23) or the Amidase_3 family (24, 25), endopeptidases (26–28), and N-acetylmuramidase (29) have been characterized to be thermostable endolysins. The Amidase_2 family endolysins Ph2119 (20) and Ts2631 (21) from *Thermus scotoductus* phages lack a CBD, and they can act on both thermophilic and mesophilic bacteria including pathogens. Besides phages, prophages are also valuable sources of endolysins, but so far only a few prophage endolysins from thermophiles have been characterized (30, 31). With the rapidly growing number of sequenced thermophile genomes, it is of great interest to exploit thermostable endolysins with antimicrobial potential from prophages of thermophiles.

*Brevibacillus thermoruber* WF146 (previously *Brevibacillus* sp. WF146 [32]) is a thermophilic bacterium with an optimum growth temperature of approximately 58°C (33). From strain WF146, three thermostable subtilases, namely, proteases WF146 (33–35), TSS (36), and BTV (37), as well as an HtrA-like protease (32) have been investigated. Meanwhile, the complete genome sequence of strain WF146 has been determined (PRJNA319752). In this study, we characterized an endolysin (LysBT1) encoded by a prophage (PBT1) in the genome of strain WF146. Recombinant LysBT1 was produced in *E. coli* BL21(DE3) and purified. LysBT1 was found to be a novel thermostable endolysin comprising a NALAA domain as the EAD and an SLH domain as the CBD, capable of acting on bacteria over a wide range of temperatures, pH values, and NaCl concentrations. The roles of Cys residues, Zn^2+^-binding residues, and the SLH domain in the stability, activity, oligomerization, and antibacterial spectrum of LysBT1 were dissected and discussed.

## RESULTS

### Prophage PBT1 of *B. thermoruber* WF146 encodes a novel endolysin (LysBT1) with a C-terminal SLH domain

**Fig 1:**
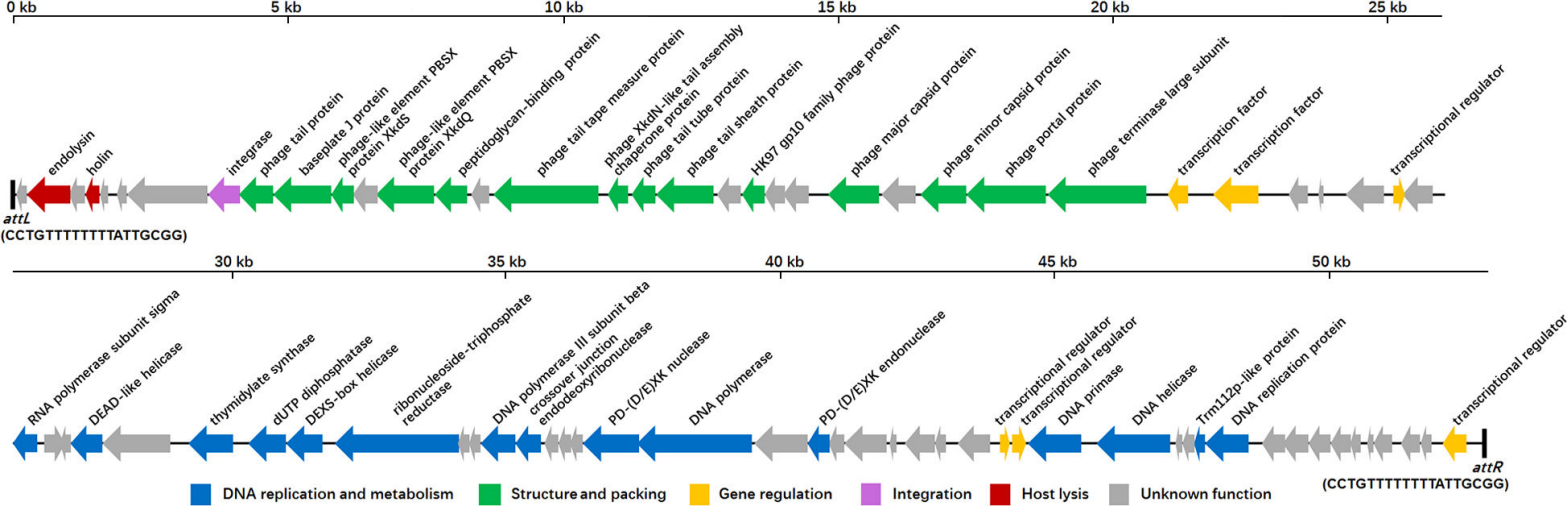
Genomic organization of the prophage PBT1. Arrows represent predicted ORFs. The sequences of *attL* and *attR* are shown.

Genome comparison analysis revealed that strain WF146 and *B. thermoruber* 423 (38) belong to the same species, with an average nucleotide identity (ANI) value of 98.76% and a 16S rRNA gene sequence identity of 99.72%, which are higher than the recommended threshold for species delineation (95% ANI and 98.7% 16S rRNA gene sequence identity) (39). The genome of strain WF146 contains a 53-kb-long (bp 3021017 to 3074038) prophage PBT1 with a total of 79 predicted open reading frames (ORFs) and two attachment sites (*attL* and *attR*) (Fig. 1).

**Fig 2:**
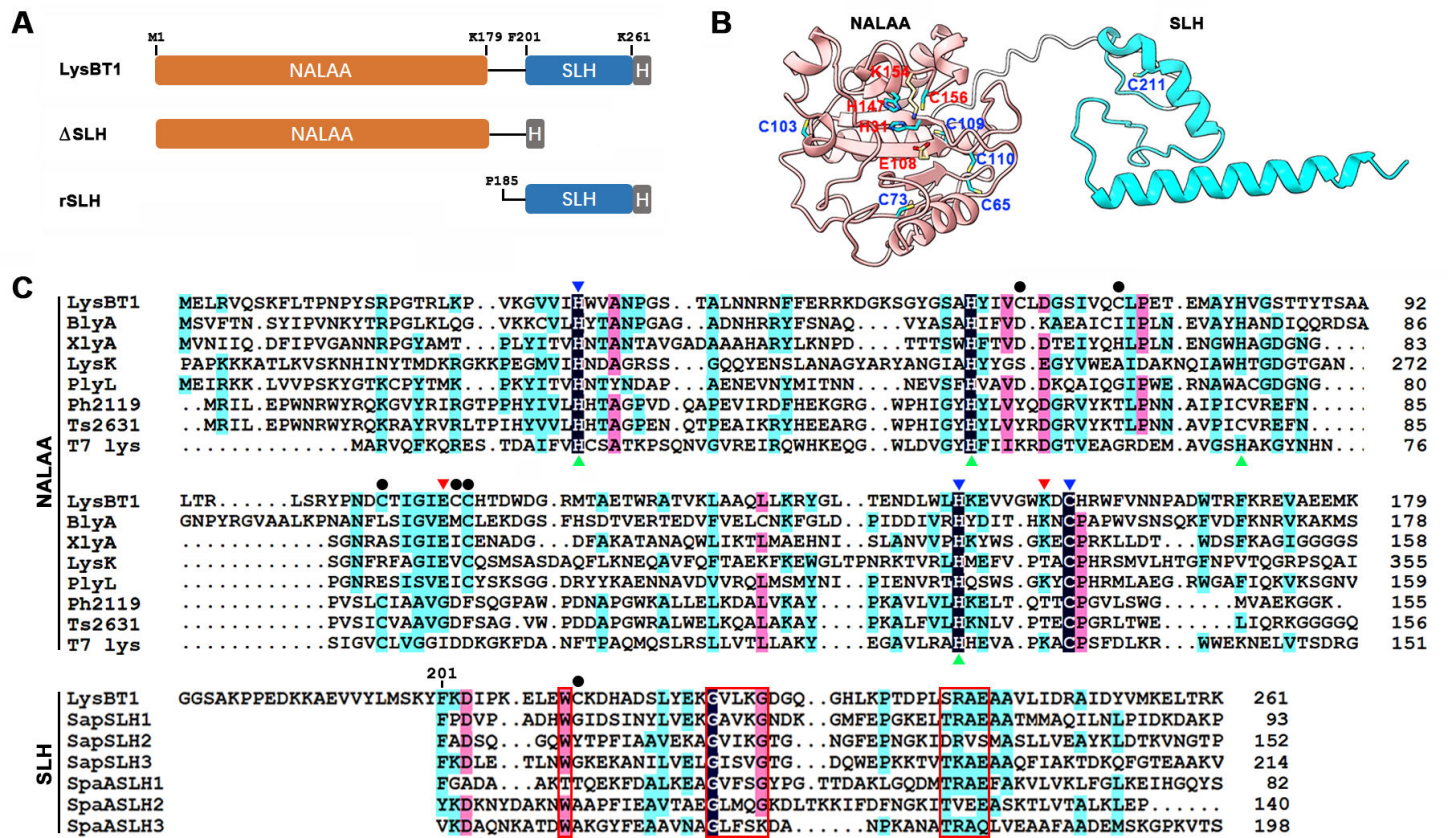
Primary and tertiary structures of LysBT1. (**A**) Schematic representation of primary structure of LysBT1 and its derivatives. The NALAA domain, the SLH domain, and the fused His-tag (H), as well as the locations of the N- and C-terminal residues of each domain, are indicated. (**B**) Structural model of LysBT1. (**C**) Amino acid sequence alignment of LysBT1 with its homologs. The NALAA domain of LysBT1 (WP_065068396) was aligned with those of BlyA (AAC38300), XlyA (P39800), LysK (YP_009041293), PlyL (AAP27798), Ph2119 (AHF20915), Ts2631 (AIM47292), and T7 lysozyme (P00806). The SLH domain in LysBT1 was aligned with those in S-layer proteins of *B. anthracis* (SapSLH1, SapSLH2, and SapSLH3; P49051) and *P. alvei* (SpaASLH1, SpaASLH2, and SpaASLH3; EJW18428). The blue and red arrowheads indicate the active site residues involved in catalytic Zn^2+^ binding and catalysis, respectively. The His residues responsible for the structural collapse of T7 lysozyme at pH values of 5.0 and lower are indicated by green arrowheads. The Cys residues except Cys156 in the active site are marked by filled circles. The conserved Trp residue, the GIIxG motif, and the TRAE motif involved in binding SCWP are boxed.

The prophage PBT1 encodes an endolysin (LysBT1) comprising an N-terminal NALAA domain and a C-terminal SLH domain (Fig. 2A and B). Phylogenetic analysis showed that LysBT1 belongs to the Amidase_2 family (Fig. S1), although its NALAA domain shows a low sequence identity (~16% to 29%) to those of known endolysins of this family, such as BlyA (10), XlyA (11), LysK (5), PlyL (12), Ph2119 (20), Ts2631 (21), and T7 lysozyme (9) (Fig. 2C). The NALAA domain of LysBT1 adopts a three-dimensional fold (Fig. 2B) similar to those of structurally characterized XlyA (11), PlyL (12), and T7 lysozyme (9). The active site residues responsible for catalytic Zn^2+^ binding and catalysis are well conserved among these enzymes (Fig. 2C). By contrast, the SLH domain of LysBT1 does not show sequence similarity to any known endolysin CBDs, but shares low sequence identity (~13-31%) with the SLH domains in S-layer proteins Sap (14, 17) and SpaA (15, 16). Meanwhile, the SLH domain of LysBT1 possesses the conserved Trp residue, the GIIxG motif, and the TRAE motif involved in the binding of Sap (14, 17) or SpaA (15, 16) to peptidoglycan-linked SCWP (Fig. 2C). Moreover, the predicted structure of the SLH domain in LysBT1 is highly similar to those in Sap (14) and SpaA (15), comprising two helices connected by an extended loop (Fig. 2B). Besides LysBT1, strain WF146 genome encodes 14 extracellular SLH domain-containing proteins with three tandem SLH domains at either the N- or C-terminus, including an S-layer protein showing ~51% sequence identity to the characterized S-layer protein of *Brevibacillus choshinensis* (40), and the SCWP-binding motifs are well conserved in these SLH domains (Fig. S2).

Compared with its homologs, LysBT1 possesses a higher content (28.4%) of charged residues (Table S1). According to the structural model of LysBT1, most of the charged residues are distributed on the enzyme surface; 54 predicted ion pairs (11 salt bridges and 43 long-range ion pairs) were found, and most of them are involved in forming ionic networks (Table S2).

### Expression of recombinant LysBT1 affects cell shape of *E. coli*

**Fig 3:**
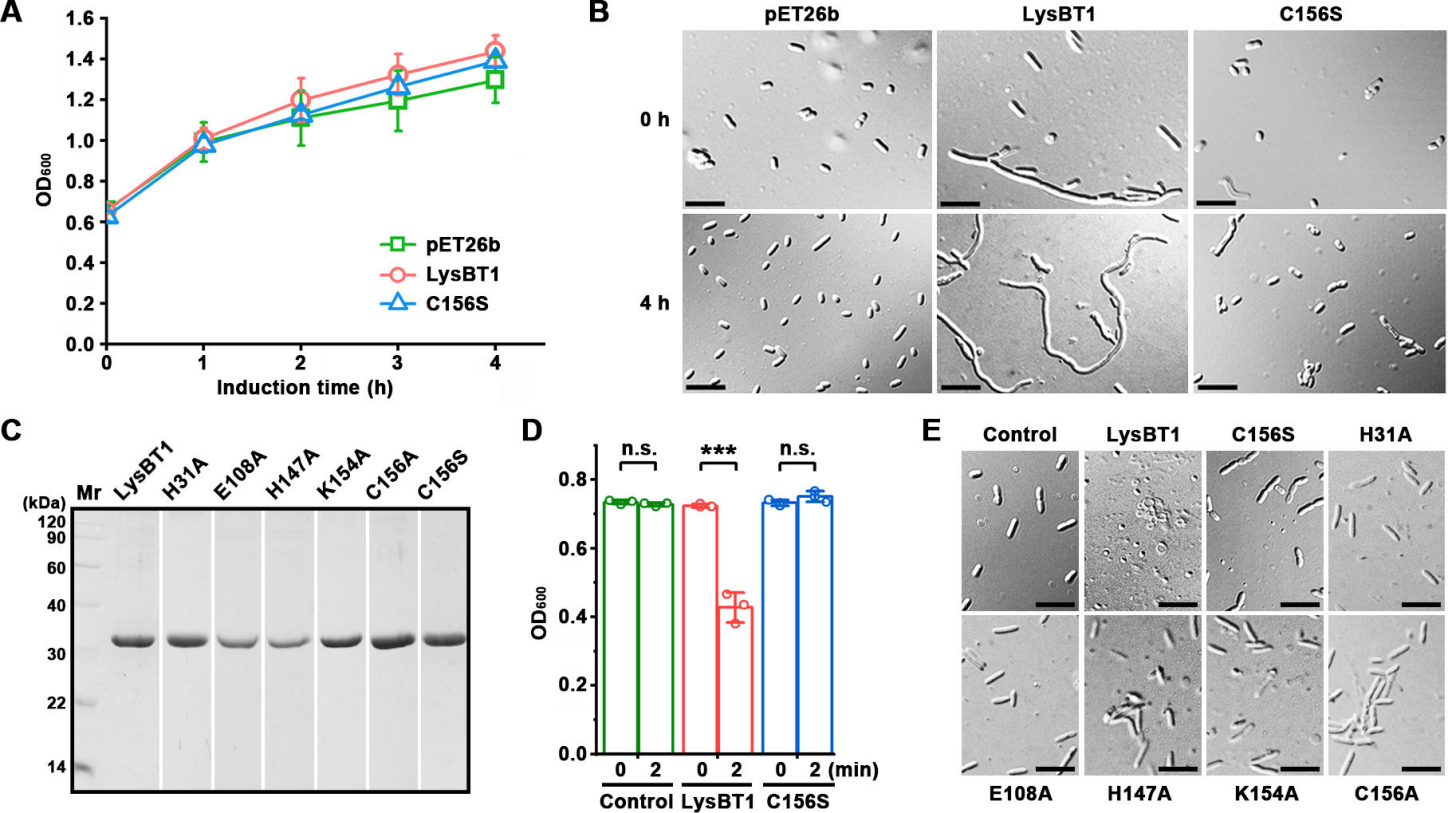
Expression of LysBT1 and its active-site variant C156S in *E. coli* BL21(DE3). (**A and B**) Growth curves and morphology of *E. coli* strains. The strains carrying pET26b and expression plasmid for target protein, respectively, were grown to an OD_600_ of 0.6–0.7 (0 h) and induced with IPTG at 30°C for different time periods; samples were subjected to OD_600_ measurement (**A**) and phase-contrast microscopy (**B**). (**C**) SDS-PAGE analysis of the purified enzymes. (**D and E**) Lysis of strain WF146 cells by LysBT1. Strain WF146 cells were incubated at 37°C in the phosphate buffer (50 mM NaH_2_PO_4_-NaOH, pH 7.4) without (control) or with the enzyme (1.32 µM) for 2 min (**D**) or with the enzyme (0.33 µM) and 0.15 M NaCl for 1 h (**E**), followed by OD_600_ measurement (**D**) and phase-contrast microscopy (**E**), respectively. Bars, 5 µm (**B and E**). The data are expressed as means ± SDs of three independent experiments (***, *P* < 0.001; n.s., no significance; calculated by Student’s *t* test) (**A and D**).

**Fig 4:**
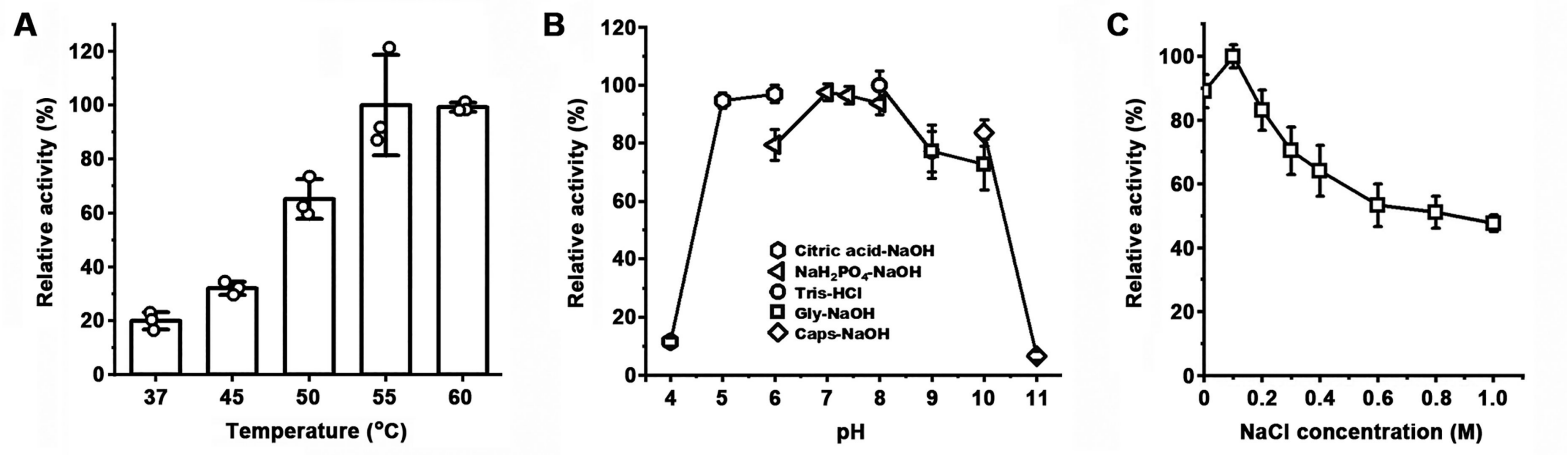
Lytic activity of LysBT1. (**A**) Temperature dependence of lytic activity. Using strain WF146 cells as the substrate, the lytic activity assay of the enzyme (0.033 µM) was carried out at different temperatures for 2 min in the phosphate buffer (50 mM NaH_2_PO_4_-NaOH, pH 7.4) containing 0.15 M NaCl. (**B**) pH dependence of lytic activity. The lytic activity assay of the enzyme (0.13 µM) was performed at 37°C for 1 h in 50 mM of different buffers (containing 0.15 M NaCl) with different pH values. (**C**) Salinity dependence of lytic activity. The lytic activity assay of the enzyme (0.33 µM) was performed at 37°C for 1 h in the phosphate buffer containing different NaCl concentrations. Relative activity was calculated by defining the highest activity among the data set (A, B, and D) as 100%. The residual activity is expressed as a percentage of the initial activity (**C**). The values are expressed as means ± SDs from three independent experiments.

The gene encoding LysBT1 with a C-terminal His-tag (Fig. 2A) and its variants, including the catalytic Zn^2+^ binding residue variants (H31A, H147A, C156A, and C156S) and the catalytic residue variants (E108A and K154A), were expressed in *E. coli* BL21(DE3). After induction with isopropyl-β-D-thiogalactopyranoside (IPTG) at 30°C, the *E. coli* strain expressing LysBT1 or the variant C156S, for example, displayed a growth curve similar to that of the control strain harboring a blank vector pET26b (Fig. 3A). However, cell shape of the strain expressing LysBT1 rather than C156S was changed to abnormal filamentous morphology (Fig. 3B, 4 h), implying that active LysBT1 affects *E. coli* cell division. Although recombinant LysBT1 lacking a signal peptide is unable to be secreted into the periplasm, it is possible that the overexpression of LysBT1 in *E. coli* may affect the stability of the inner membrane, leading to the release of the endolysin into the periplasm to hydrolyze the peptidoglycan. Additionally, we observed that the culture of the LysBT1-expressing *E. coli* strain took a longer time (~5 h) than the C156S-expressing strain or the control strain (~2 h) to reach an optical density at 600 nm (OD_600_) of 0.6–0.7 before IPTG induction (Fig. S3). Moreover, some cells of the LysBT1-expressing *E. coli* strain became filamentous even before IPTG induction (Fig. 3B, 0 h), most likely due to a low basal expression of recombinant protein in *E. coli* BL21(DE3) in the absence of the inducer (41). We speculated that active LysBT1 in the cytoplasm may act on peptidoglycan precursors with a stem peptide (e.g., Lipid II, Lipid I, and UDP-MurNAc-pentapeptide) (42) to affect peptidoglycan synthesis and cell division.

Recombinant LysBT1 and its active-site variants were present in both soluble and insoluble fractions of *E. coli* cells and were purified from the soluble fractions (Fig. 3C). The incubation of *B. thermoruber* WF146 cells at 37°C with purified LysBT1 but not C156S led to a decrease of cell density (Fig. 3D). Meanwhile, C156S-treated strain WF146 cells remained as rod-shaped forms, while LysBT1-treated cells converted from rod-shaped forms to spheres or cell debris (Fig. 3E). Similar to the case of C156S, the incubation of strain WF146 cells with other active-site variants (H31A, E108A, H147A, K154A, and C156A) at 37°C did not lead to a remarkable decrease in cell density (Fig. S4) or a change in cell morphology (Fig. 3E). These results confirm that recombinant LysBT1 has the ability to hydrolyze the peptidoglycan of strain WF146, and the residues responsible for catalytic Zn^2+^ binding and catalysis are necessary for the lytic activity of LysBT1.

### LysBT1 is highly thermostable and resistant to polyextreme conditions

LysBT1 exhibited a higher lytic activity against the exponential phase cells than the stationary phase cells of strain WF146 (Fig. S5), probably due to the accumulation of chemical modifications and thickening of peptidoglycan in the stationary phase. Hereafter, the exponential phase cells were used as the substrates for measuring the lytic activity of the enzyme. LysBT1 showed an increased lytic activity with the increase of temperature from 37°C to 55°C (Fig. 4A). A quantitative comparison of the lytic activity of LysBT1 at 65°C or higher temperatures was hampered by substantial cell lysis occurring at these elevated temperatures even in the absence of the enzyme (Fig. S6). LysBT1 displayed relatively high activity (>70%) at pH 5.0–10.0 and 37°C, with the highest activity at pH 5.0–8.0 (Fig. 4B). Microscopy analysis confirmed the lytic activity of LysBT1 against strain WF146 cells over the pH range of 4.0–11.0 (Fig. S7). Meanwhile, LysBT1 is highly stable in a wide pH range, retaining more than 70% activity at pH 4.0–11.0 and more than 80% activity at pH 5.0–10.0 after incubation at 37°C for 1 h and 6 h, respectively (Fig. S8). LysBT1 showed the highest activity around 0.1 M NaCl and retained ~50% activity at 1 M NaCl (Fig. 4C).

Although LysBT1 contains the conserved residues (H31, H147, and C156) coordinating the catalytic Zn^2+^ (Fig. 1C), its lytic activity was not affected by the addition of 1 mM EDTA or phenanthroline at 37°C (Table 1). Moreover, the 50 mM EDTA-treated LysBT1 exhibited a similar level of activity to the untreated enzyme (Table 1), suggesting that LysBT1 is highly resistant to metal chelators. In addition, the lytic activity of LysBT1 could be improved in the presence of H_2_O_2_ (0.5%–2.5%) or β-mercaptoethanol (β-Me; 0.5%–2.5%; Table 1), presumably reflecting a synergistic effect of peptidoglycan hydrolysis by LysBT1 and cell envelope damage caused by the oxidant or reductant. With the exception of Mg^2+^, all the tested divalent metal ions (1 mM) including Zn^2+^ inhibited the activity of the untreated or EDTA-treated LysBT1, with Fe^2+^ being the strongest inhibitor (Table 1). Consistent with this, microscopy analysis also revealed the strongest inhibitory effect of Fe^2+^ on lytic activity of LysBT1 (Fig. S9). We noticed that the untreated LysBT1 was inhibited to a greater extent than that of the EDTA-treated enzyme by the same divalent metal ion (Table 1), raising the possibility that the untreated LysBT1 contains some metal ions that affect enzyme stability and activity but can be removed by EDTA.

**TABLE 1:** Effects of metal ions, chelators, and redox agents on lytic activity of LysBT1

Agent	Concentration	Relative activity (%)^*a*^	
		LysBT1	EDTA-treated LysBT1
None		100.0 ± 7.7	110.5 ± 5.3
MgCl_2_	1 mM	95.6 ± 10.8	98.0 ± 9.4
CaCl_2_	1 mM	50.7 ± 6.0	59.8 ± 6.2
FeCl_2_	1 mM	1.4 ± 1.3	6.7 ± 0.2
ZnCl_2_	1 mM	17.1 ± 4.2	43.7 ± 10.2
CoCl_2_	1 mM	46.6 ± 13.4	67.4 ± 9.3
CuCl_2_	1 mM	19.7 ± 18.6	42.8 ± 24.8
EDTA	1 mM	100.9 ± 3.5	104.3 ± 4.9
Phenanthroline	1 mM	120.9 ± 3.1	n.d.
β-Me	0.5%	130.09 ± 9.54	n.d.
	2.5%	154.49 ± 11.84	n.d.
Dithiothreitol	1%	110.7 ± 7.3	n.d.
H_2_O_2_	0.5%	149.02 ± 4.43	n.d.
	2.5%	152.21 ± 12.36	n.d.

^*a*^ Using strain WF146 cells as the substrate, the lytic activity of LysBT1 or EDTA-treated LysBT1 (0.33 µM) was determined at 37°C for 1 h in the Tris buffer (50 mM Tris-HCl, pH 7.4) containing the indicated agent. The EDTA-treated LysBT1 was prepared by incubating the enzyme with 50 mM EDTA at room temperature for 1 h in the Tris buffer, followed by dialysis against the Tris buffer at 4°C overnight. The relative activity was calculated by defining the activity of LysBT1 without additives as 100%. The values are expressed as means ± SDs from three independent experiments. n.d., not determined.

**Fig 5:**
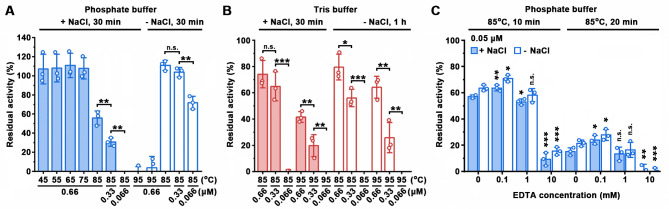
Effects of temperature (**A and B**), enzyme concentration (**A and B**), buffer system (**A and B**), and EDTA (**C**) on the thermostability of LysBT1. Different concentrations of LysBT1 (0.05–0.66 μM) were incubated at different temperatures for the indicated time periods in the phosphate buffer (50 mM NaH_2_PO_4_-NaOH, pH 7.4) or the Tris buffer (50 mM Tris-HCl, pH 7.4) without (−) or with (+) 0.15 M NaCl and/or different concentrations of EDTA, followed by activity assay performed at 37°C for 1 h using the cells (**A and B**) or isolated peptidoglycan (**C**) of strain WF146 as the substrate. The residual activity is expressed as a percentage of the initial activity. The data are expressed as means ± SDs of three independent experiments (***, *P* < 0.001; **, *P* < 0.01; *, *P* < 0.05; n.s., no significance; calculated by Student’s *t* test). In panel C, the significant differences between the samples without EDTA and those with different concentrations of EDTA were calculated.

The thermostability of LysBT1 was investigated by measuring the residual activity of the enzyme incubated under various conditions. After incubation at different temperatures for 30 min in the phosphate buffer with 0.15 M NaCl, LysBT1 was stable at temperatures up to 75°C and retained 55.9% residual activity at 85°C (Fig. 5A). The enzyme did not show any loss of activity after incubation at 85°C for 30 min when NaCl was omitted from the buffer (Fig. 5A). These results suggest that LysBT1 is highly thermostable, and NaCl negatively affects the thermostability of the enzyme.

We found that the type of buffer system influences the thermostability of LysBT1. For instance, regardless of the presence or absence of NaCl, LysBT1 (0.66 µM) was almost completely inactivated after incubation at 95°C for 30 min in the phosphate buffer (Fig. 5A). By contrast, LysBT1 retained 41.6% and 64.2% residual activity after incubation in the Tris buffer at 95°C for 30 min in the presence of NaCl and for 1 h in the absence of NaCl, respectively (Fig. 5B). Notably, the thermostability of LysBT1 showed a trend of increasing with the increase of enzyme concentration, in either the presence or absence of NaCl (Fig. 5A). This trend still exists when the phosphate buffer was replaced by the Tris buffer (Fig. 5B).

The effect of EDTA on the thermostability of LysBT1 was also investigated. The residual activity of the heat-treated enzyme was determined using the isolated peptidoglycan instead of the cells of strain WF146 as the substrate, in order to exclude the possibility that EDTA affects the cells and, in turn, disturbs the activity assay. Compared with that without extra addition of EDTA, the residual activity of LysBT1 increased significantly at 0.1 mM EDTA but decreased at 10 mM of the chelator after incubation at 85°C for 10–20 min, in either the presence or absence of NaCl (Fig. 5C).

### The Cys residues are involved in maintaining the stability and activity of LysBT1

**Fig 6:**
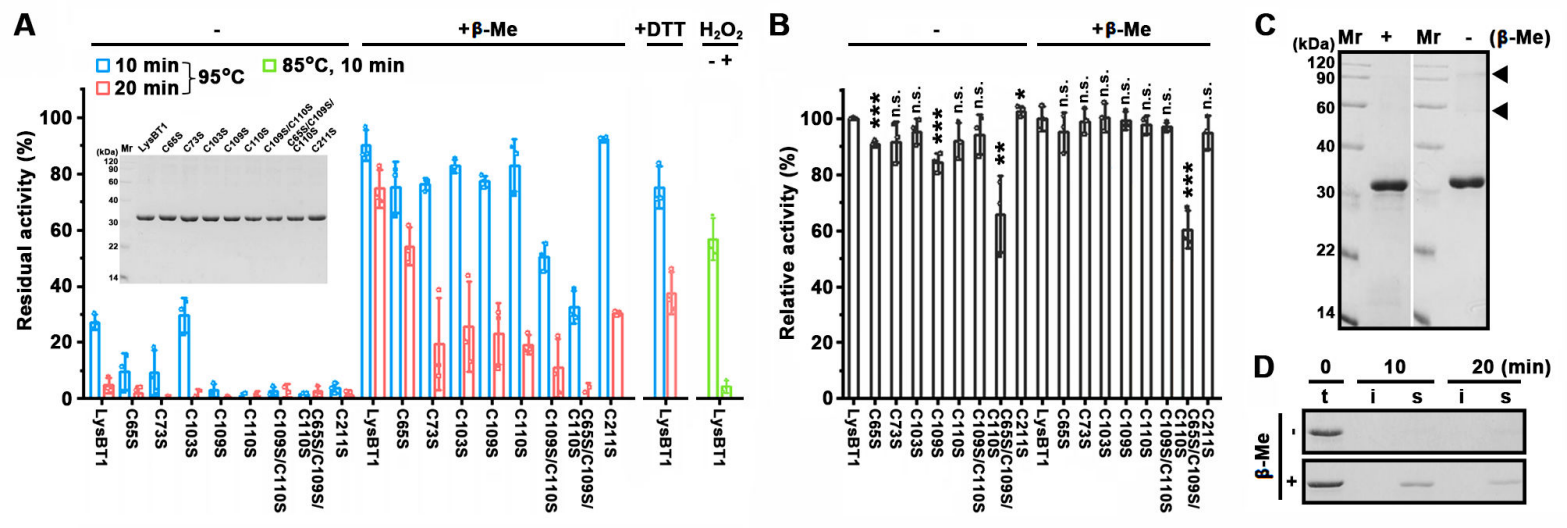
The roles of Cys residues in stability and activity of LysBT1. (**A**) Enzyme stability under non-reducing and reducing conditions. LysBT1 and its variants (0.066 µM) were incubated at 95°C or 85°C for different time periods in the Tris buffer (50 mM Tris-HCl, pH 7.4) without (−) or with (+) 0.5% of β-Me, dithiothreitol (DTT), or H_2_O_2_, followed by activity assay at 37°C for 1 h. The residual activity is expressed as a percentage of the initial activity. The inset depicts SDS-PAGE analysis of purified enzymes. (**B**) Lytic activity under non-reducing and reducing conditions. The lytic activity assay of the enzyme (0.033 µM) was performed at 37°C for 1 h in the Tris buffer without (−) or with (+) 0.5% β-Me. Relative activity was calculated by defining the activity of LysBT1 in the two groups (without or with β-Me) as 100%, respectively. (**C**) SDS-PAGE analysis of purified LysBT1 in the absence (−) or presence (+) of β-Me. Arrowheads indicate the disulfide bond cross-linked forms of LysBT1. (**D**) Heat resistance of C156S. The enzyme (0.066 µM) was incubated at 95°C in the Tris buffer without (−) or with (+) 0.5% β-Me. At the indicated time intervals, samples (t) were withdrawn, and the soluble (s) and insoluble (i) fractions were separated by centrifugation, followed by SDS-PAGE analysis. The data are expressed as means ± SDs of three independent experiments (***, *P* < 0.001; **, *P* < 0.01; *, *P* < 0.05; n.s., no significance; calculated by Student’s *t* test).

LysBT1 contains seven Cys residues including the catalytic Zn^2+^-coordinating Cys156, but none of them are involved in intramolecular disulfide bond formation according to the structural model of the enzyme (Fig. 2B). Meanwhile, only minor intermolecular disulfide bond cross-linked forms of LysBT1 were detected by SDS-PAGE analysis under non-reducing condition (Fig. 6C). To investigate the individual and possible cumulative roles of Cys residues in enzyme stability and activity, we constructed seven single-site variants (C65S, C73S, C103S, C109S, C110S, C156S, and C211S), a double-site variant C109S/C110S, and a triple-site variant C65S/C109S/C110S, compared with LysBT1, C65S, C73S, C109S, C110S, and C211S but not C103S retained lower residual activity after incubation at 95°C for 10–20 min (Fig. 6A). A 10 min incubation at 95°C led to complete degradation of the inactive variant C156S (Fig. 6D), most likely via thermal hydrolysis (43). These results suggest that six of the seven Cys residues contribute to the thermostability of LysBT1, although they do not form stabilizing disulfide bonds.

LysBT1 retained higher residual activity after incubation with β-Me or DTT at 95°C than in the absence of a reducing agent, whereas the presence of H_2_O_2_ decreased the stability of the enzyme at 85°C (Fig. 6A). This indicates that reducing agents could protect LysBT1 against oxidative destabilization. Similar to LysBT1, the Cys residue variants retained higher residual activity or protein quantity following heat treatment at 95°C in the presence of β-Me than in the absence of the reducing agent (Fig. 6A and D). Moreover, in the presence of β-Me C65S/C109S/C110S was less heat resistant than C109S/C110S, which was less heat resistant than the single-site variants (C65S, C109S, and C110S; Fig. 6A), reflecting the cumulative roles of Cys residues in stabilizing LysBT1 against oxidative damage. The variants C65S, C109S, and C65S/C109S/C110S exhibited lower activity than LysBT1 in the absence and/or presence of β-Me at 37°C (Fig. 6B), possibly because Cys65, Cys109, and Cys110 are spatially close to the catalytic residue Glu108 (Fig. 2B), and thus, their mutations affect enzyme catalysis.

### The SLH domain contributes to the stability, activity, oligomerization, and cell-wall binding ability of LysBT1

**Fig 7:**
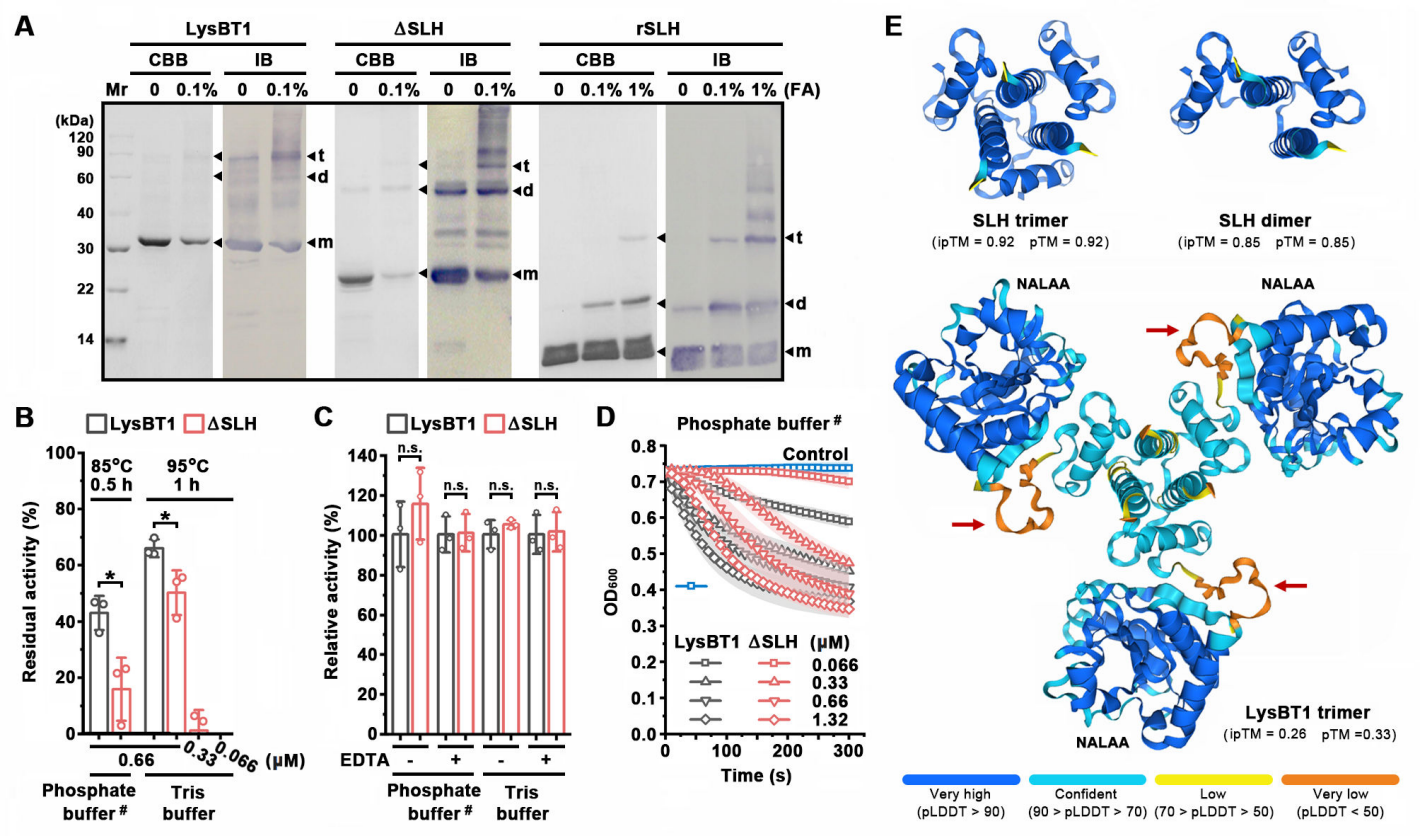
Roles of the SLH domain in stability, activity, and oligomerization of LysBT1. (**A**) Oligomerization capacities of LysBT1 and its derivatives. Purified LysBT1, ΔSLH, rSLH, and the samples cross-linked with 0.1% or 1% glutaraldehyde (FA) were subjected to SDS-PAGE (stained with Coomassie brilliant blue [CBB]) or anti-His tag immunoblot (IB) analyses. The arrows indicate the positions of monomer (m), dimer (d), and trimer (t). (**B**) Thermostabilities of the enzymes. LysBT1 (0.66 µM) and ΔSLH (0.066–0.66 µM) were incubated at 85°C or 95°C for the indicated time periods in the phosphate buffer (50 mM NaH_2_PO_4_-NaOH, pH 7.4) containing 0.15 M NaCl (marked by “#”) or the Tris buffer (50 mM Tris-HCl, pH 7.4), followed by lytic activity assay using strain WF146 cells as the substrate. The residual activity is expressed as a percentage of the initial activity. (**C**) Comparison of lytic activities of LysBT1 and ΔSLH against strain WF146 cells. The lytic activity assay of the enzyme (0.33 µM) was performed at 37°C for 1 h in the NaCl-containing phosphate buffer (marked by “#”) or the Tris buffer without (−) or with (+) 1 mM EDTA. Relative activity was calculated by defining the activity of LysBT1 under each of the conditions as 100%. (**D**) The Iinitial stage of bacteriolytic reaction catalyzed by LysBT1 or ΔSLH. Strain WF146 cells were incubated alone (control) or with different concentrations of the enzyme at 37°C, and the change in OD_600_ of the reaction mixture was recorded over time. (**E**) Predicted structures of SLH trimer, SLH dimer, and LysBT1 trimer. Red arrows indicate the flexible linker regions between the NALAA and SLH domains. The data are expressed as means ± SDs of three independent experiments (*, *P* < 0.05; n.s., no significance; calculated by Student’s *t* test; **B and C**).

To investigate the function of the SLH domain of LysBT1, we constructed the SLH domain-deletion variant (ΔSLH) and the recombinant SLH domain (rSLH; Fig. 2A), and the recombinant proteins expressed in *E. coli* BL21(DE3) were purified (Fig. 7A). Regardless of the type of buffer system used for the experiments, ΔSLH retained a lower residual activity than LysBT1 after heat treatment at 85°C or 95°C (Fig. 7B), suggesting a stabilizing role of the SLH domain in LysBT1. Similar to LysBT1, ΔSLH displayed an increase of thermostability with the increase of enzyme concentration (Fig. 7B).

LysBT1 and ΔSLH exhibited a similar level of activity (determined by a single time-point assay at 37°C for 1 h) against strain WF146 cells in the absence or presence of EDTA (Fig. 7C), suggesting that the SLH domain is not necessary for LysBT1 to lyse strain WF146 cells. Nevertheless, in contrast to the case of LysBT1, the reaction progress curve of ΔSLH at 37°C showed a lag phase at the initial stage, and the lag phase shortened as the ΔSLH concentration increased (Fig. 7D), reflecting a slow substrate-binding step because the SLH domain can mediate the binding of LysBT1 to the cell surface (see below).

The formaldehyde cross-linking experiments showed that LysBT1 could form a trimer, although a minor amount of dimeric forms were detected (Fig. 7A). ΔSLH and rSLH could also form dimers and trimers (Fig. 7A). In Sap or SpaA, three tandem SLH domains fold into a three-prong spindle-like pseudotrimer (42, 44). The structural prediction of the three tandem SLH domains in each of the 14 SLH domain-containing proteins of strain WF146 (Fig. S2) revealed a similar pseudotrimer (Fig. S10). Similarly, the SLH trimer of LysBT1 was predicted to adopt a structure of three-prong spindle with high interface predicted template modeling (ipTM) and predicted template modeling (pTM) scores (0.92; Fig. 7E), suggesting a confident high-quality prediction (44). The SLH domain of LysBT1 was predicted to be capable of forming a dimer as well, in which the two SLH domains are arranged in a similar manner to that in the SLH trimer (Fig. 7E). It appears that the dimer represents an intermediate species during trimerization of the SLH domains. In contrast to the SLH trimer and dimer, the predicted structures of ΔSLH trimer and dimer showed lower ipTM and pTM scores (≤0.5; Fig. S11). In the predicted structure of LysBT1 trimer, SLH domains mediate the trimerization of the enzyme, and each SLH domain is connected to a NALAA domain through a flexible linker (Fig. 7E). Nine salt bridges and three long-range ion pairs were predicted at the interfaces between SLH domains in LysBT1 trimer (Table S2). The ipTM and pTM scores of the predicted structure of LysBT1 trimer are low (<0.5), mainly due to the very low pLDDT (a per-residue confidence metric) value (https://golgi.sandbox.google.com/) of the flexible linker region, which may allow the NALAA domains in the LysBT1 trimer to move independently and facilitate them to access and act on the substrates.

**Fig 8:**
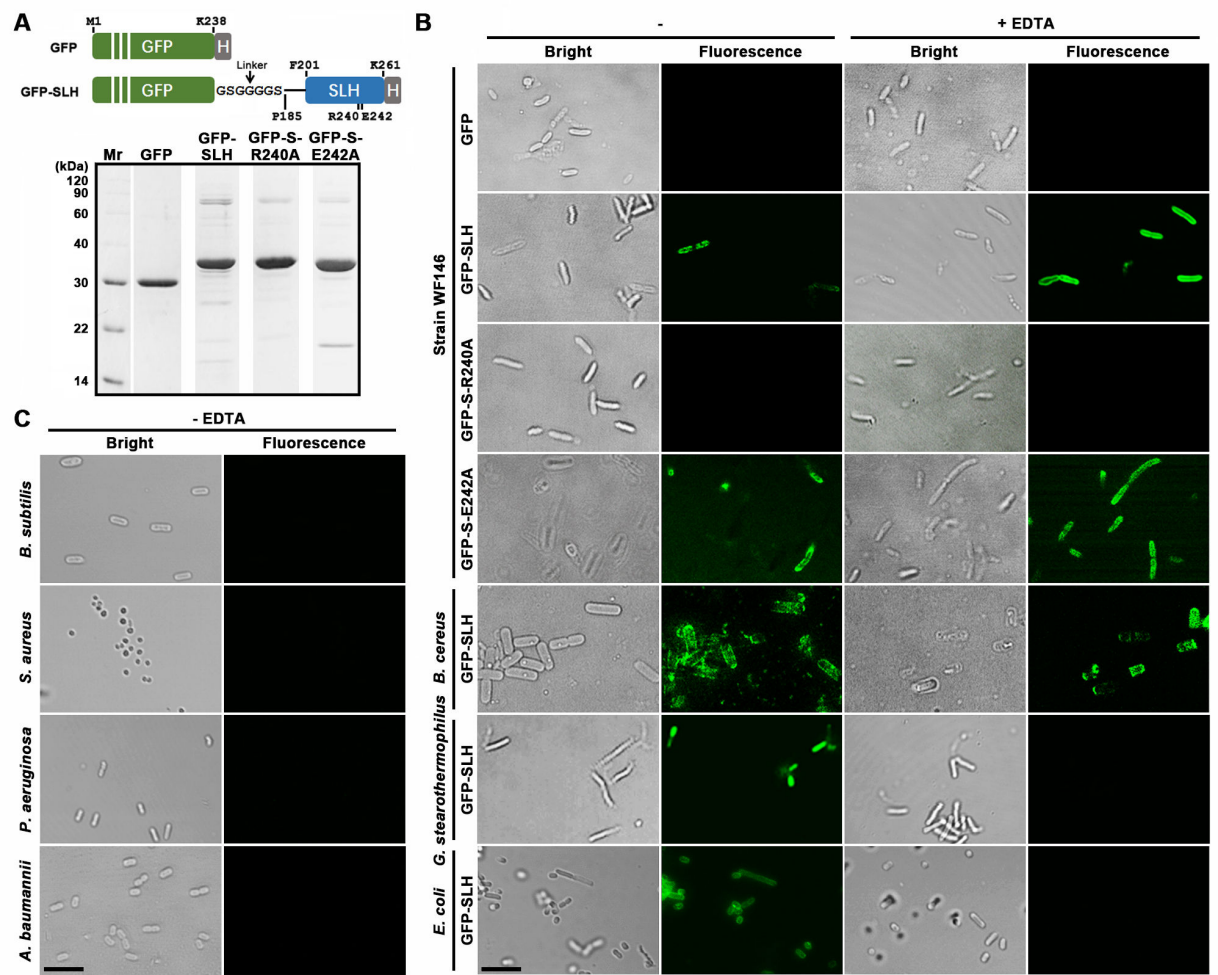
Bacterial cell surface-binding capacity of the SLH domain of LysBT1. (**A**) SDS-PAGE analysis of the purified proteins. Schematic representation of the primary structure of recombinant GFP and GFP-SLH are shown on the top of this panel. The SLH domain, the fused His-tag (H), the GFP domain, the fused linker, the locations of the N- and C-terminal residues of each domain, as well as the residues R240 and E242 in the TRAE motif of the SLH domain are indicated. (**B and C**) Cell surface-binding capacity of the SLH domain. Bacterial cells were incubated with recombinant GFP, GFP-SLH, or its derivatives (0.16 µM) at 37°C for 1 h in the Tris buffer (50 mM Tris-HCl, pH 7.4) without (−) or with (+) 10 mM EDTA, washed with the same buffer, and examined by phase-contrast and fluorescence microscopy. Bars, 5 µm.

To investigate the cell wall-binding capacity of the SLH domain of LysBT1, the GFP-tagged SLH domain (GFP-SLH) and its derivatives (GFP-S-R240A and GFP-S-E242A) with mutations in the TRAE motif (Fig. 2C) were expressed and purified from *E. coli* BL21(DE3) (Fig. 8A). Fluorescence microscopy images revealed that GFP-SLH and GFP-S-E242A could bind to the cell surface of strain WF146, whereas GFP-S-R240A lost the binding capacity toward strain WF146 cell (Fig. 8B). These results suggest that the SLH domain has the ability to mediate the binding of LysBT1 to strain WF146 cell wall and confirm the importance of Arg240 of the TRAE motif in ligand binding. The presence of EDTA appears to improve the cell binding capacity of GFP-SLH or GFP-S-E242A, leading to a relatively large number of fluorescently labeled cells (Fig. 8B). This is probably because EDTA-induced release of S-layer proteins from the cell wall facilitates the binding of the GFP-tagged SLH domains to the cell.

Besides strain WF146, GFP-SLH could bind to Gram-positive *Geobacillus stearothermophilus* and *Bacillus cereus*, while the presence of EDTA lead to the loss of cell binding ability of GFP-SLH to *G. stearothermophilus* rather than *B. cereus* (Fig. 8B). These results suggest that the binding ligand of the SLH domain could be removed from *G. stearothermophilus* by EDTA. In the case of *B. cereus*, weak fluorescence signals were detected at some sites without intact cells, probably due to the binding of GFP-SLH to a minor amount of cell debris generated during sample preparation (Fig. 8B). Interestingly, GFP-SLH was able to bind to Gram-negative *E. coli* BL21(DE3) of serotype O_7_:H^−^ in the absence but not presence of EDTA capable of disrupting OM (Fig. 8B), implying that the binding ligand of the SLH domain localizes on the surface of OM rather than within the peptidoglycan of *E. coli* BL21(DE3). Other tested bacteria including *B. subtilis* DB104, *S. aureus*, *Pseudomonas aeruginosa*, and *Acinetobacter baumannii* were not bound by GFP-SLH, in either the absence (Fig. 8C) or presence of EDTA (Fig. S12), suggesting that the cell wall or cell envelope of these bacteria lacks the binding ligand of the SLH domain of LysBT1.

### Lytic activity of LysBT1 against Gram-positive and Gram-negative bacteria

Among the tested Gram-positive bacteria, in addition to strain WF146, *G. stearothermophilus* and *B. subtilis* DB104 showed decreased cell viability after treatment with LysBT1 or ΔSLH alone at 37°C for 1 h, whereas *B. cereus* and *S. aureus* were unsusceptible to either LysBT1 or ΔSLH under the same conditions (Table 2). Nevertheless, the treatment of *B. cereus* by LysBT1 or ΔSLH with EDTA led to a decrease in cell viability (Table 2). We noticed that although LysBT1 could not reduce the viability of *B. cereus* in the absence of EDTA (Table 2), the SLH domain was able to bind to this bacterium under the same condition (Fig. 8B), reflecting the difference between the SLH-mediated binding spectrum and the antibacterial spectrum of LysBT1.

**TABLE 2:** Antibacterial activity of LysBT1, ΔSLH, HEWL, and polymyxin against different bacteria

Strain	Additive	Log CFU/mL^*a*^					
		Control	LysBT1 0.33 µM	ΔSLH 0.33 µM	HEWL 0.33 µM	Polymyxin	
						0.33 µM	0.33 mM
*G. stearothermophilus*	–^*b*^	8.30 ± 0.30	4.35 ± 0.38 (***)	4.46 ± 0.33 (***)	0 (****)	8.22 ± 0.26 (n.s.)	0 (****)
*B. subtilis* DB104	–	8.28 ± 0.11	7.29 ± 0.09 (***)	7.19 ± 0.16 (***)	7.18 ± 0.16 (***)	7.78 ± 0.11 **	6.32 ± 0.38 (**)
*S. aureus*	–	8.56 ± 0.28	8.57 ± 0.19 (n.s.)	8.66 ± 0.23 (n.s.)	ND	ND	ND
*B. cereus*	–	7.48 ± 0.19	7.50 ± 0.15 (n.s.)	7.43 ± 0.13 (n.s.)	6.96 ± 0.31 (n.s.)	7.16 ± 0.22 (n.s.)	0.85 ± 0.01 (****)
	1 mM EDTA	7.17 ± 0.11	6.82 ± 0.08 (*)	6.73 ± 0.08 (**)	5.98 ± 0.36 (**)	6.97 ± 0.34 (n.s.)	0.84 ± 0.02 (****)
*E. coli* BL21(ED3)	–	8.74 ± 0.11	8.46 ± 0.05 (*)	8.73 ± 0.08 (n.s.)	8.69 ± 0.11 (n.s.)	8.89 ± 0.17 (n.s.)	0 (****)
	1 mM EDTA	8.80 ± 0.12	7.36 ± 0.32 (**)	8.39 ± 0.07 (**)	6.68 ± 0.12 (****)	8.80 ± 0.06 (n.s.)	0 (****)
	5 mM EDTA	8.76 ± 0.02	8.25 ± 0.21 (*)	8.11 ± 0.33 (*)	ND	ND	ND
	10 mM EDTA	8.72 ± 0.07	7.87 ± 0.12 (***)	8.48 ± 0.13 (*)	ND	ND	ND
	5 mM citric acid	8.64 ± 0.18	7.82 ± 0.30 (*)	7.90 ± 0.19 (**)	ND	ND	ND
	10 mM citric acid	7.30 ± 0.41	4.76 ± 1.02 (*)	5.55 ± 0.54 (*)	ND	ND	ND
*A. baumannii*	–	8.85 ± 0.15	8.88 ± 0.13 (n.s.)	9.04 ± 0.03 (n.s.)	8.79 ± 0.10 (n.s.)	8.78 ± 0.20 (n.s.)	8.75 ± 0.19 (n.s.)
	1 mM EDTA	8.87 ± 0.17	8.62 ± 0.06 (n.s.)	7.83 ± 0.20 (**)	ND	ND	ND
	5 mM EDTA	8.51 ± 0.18	8.52 ± 0.06 (n.s.)	8.64 ± 0.12 (n.s.)	ND	ND	ND
	10 mM EDTA	8.66 ± 0.08	8.57 ± 0.13 (n.s.)	8.72 ± 0.09 (n.s.)	ND	ND	ND
	5 mM citric acid	8.93 ± 0.02	6.39 ± 0.21 (****)	8.40 ± 0.32 (*)	8.89 ± 0.05 (n.s.)	8.98 ± 0.08 (n.s.)	0 (****)
	10 mM citric acid	5.16 ± 0.10	4.54 ± 0.05 (***)	4.90 ± 0.18 (n.s.)	ND	ND	ND

^*a*^ Bacterial cells were incubated at 37°C for 1 h in the absence (control) or presence of the enzyme or antibiotic in the Tris buffer (50 mM Tris-HCl, pH 7.4) without (−) or with different concentrations of EDTA or citric acid, and then spotted at 1:10 serial dilution on solid LB media for cell viability assay. The data are expressed as means ± SDs of three independent experiments. Statistically significant differences between the control and experimental samples were calculated by Student’s *t* test (****, *P* < 0.0001; ***, *P* < 0.001; **, *P* < 0.01; *, *P* < 0.05; n.s., no significance). ND, not determined; HEWL, hen egg-white lysozyme.

^*b*^ –, no additive was added to the reaction mixture.

In the presence of the OM permeabilizer EDTA (1–10 mM) or citric acid (5–10 mM), LysBT1 could act on Gram-negative *E. coli* BL21(DE3) at 37°C, resulting in a decrease in cell viability (Table 2). The presence of citric acid but not EDTA also enabled LysBT1 to reduce the cell viability of *A. baumannii* at 37°C, although the addition of higher concentration (10 mM) of citric acid alone to the bacterial suspension caused a remarkable decrease in *A. baumannii* viability (Table 2). Compared with *E. coli* and *A. baumannii*, *P. aeruginosa* showed a more pronounced reduction in cell viability when incubated with EDTA or citric acid alone, but it was unsusceptible to LysBT1 even in the presence of the OM permeabilizers (Table S3). These results suggest that OM permeabilizers facilitate LysBT1 to access and hydrolyze the peptidoglycan of certain Gram-negative bacteria, and the cell lytic efficiency of the endolysin depends on both the bacterial species and the type of OM permeabilizer.

LysBT1 and ΔSLH displayed different antibacterial spectra against Gram-negative bacteria under different conditions. For instance, LysBT1 reduced the *E. coli* BL21(DE3) viability more pronouncedly than ΔSLH in the presence of 1 or 10 mM of EDTA, while the incubation with 1 mM of EDTA rendered *A. baumannii* susceptible to ΔSLH but not LysBT1 at 37°C (Table 2). Additionally, LysBT1 exhibited a higher bactericidal activity against *A. baumannii* than ΔSLH in the presence of 5 mM of citric acid (Table 2). These results demonstrate that the SLH domain is not strictly linked to antibacterial spectrum of LysBT1.

The antibacterial activity of LysBT1 was compared with those of hen egg-white lysozyme (HEWL) and the bactericidal antibiotic polymyxin. At the same enzyme concentration (0.33 µM), HEWL displayed a higher antibacterial activity against *G. stearothermophilus* or *E. coli* BL21(DE3) than LysBT1 at 37°C (Table 2). However, LysBT1 but not HEWL could reduce the viability of *A. baumannii* with the aid of 5 mM of citric acid (Table 2). When applied at the same molar concentration (0.33 µM) as that of LysBT1, polymyxin could not reduce the viability of some bacteria that are susceptible to the endolysin under certain conditions, such as *G. stearothermophilus*, *B. cereus*, *E. coli* BL21(DE3), and *A. baumannii* (Table 2). Nevertheless, these four bacteria were highly susceptible to a high concentration (0.33 mM) of polymyxin (Table 2).

## DISCUSSION

### Enzymatic property

NALAAs are Zn^2+^-dependent enzymes (7, 8), and the lytic activities of thermophilic phage-derived NALAAs, such as Ts2631 (21), PhiKo (23), PlyGVE2 (24), and LysGR1 (25), are inhibited by EDTA. By contrast, LysBT1 is resistant to EDTA. EDTA resistance has been previously described for some mesophilic phage-derived NALAAs belonging to the Amidase_2 family (45–47). One possible explanation for the EDTA resistance of these enzymes is that Zn^2+^ is not essential for their activities. However, the two His residues and one Cys residue that coordinate the catalytic Zn^2+^ in Amidase_2 family members are conserved in these EDTA-resistant NALAAs. Moreover, our results reveal that the catalytic Zn^2+^-coordinating residue variants (H31A, H147A, C156A, and C156S) of LysBT1 lose lytic activity, confirming the necessity of the catalytic Zn^2+^-coordinating residues in catalysis of NALAAs. In this context, a more likely scenario is that these EDTA-resistant NALAAs possess a high-affinity Zn^2+^-binding site, to which the catalytic Zn^2+^ binds too tightly to be removed by EDTA. Moreover, Zn^2+^ supplementation inhibits the activity of LysBT1, and a similar inhibitory effect of Zn^2+^ has been described for other endolysins with an Amidase_2 domain (45–47), possibly because non-specific binding of excess Zn^2+^ ions to the enzyme affects enzyme function.

Besides EDTA resistance, LysBT1 is also highly resistant to acidic conditions. Only a few endolysins of the Amidase_2 family (48–50) exhibit high activity at pH values of 5.0 and lower. It has been proposed that four His residues form a cluster in T7 lysozyme and will generate a repulsive force upon protonation below pH 6.0, leading to a structural collapse (51). We noticed that the four His residues of T7 lysozyme are conserved in LysBT1 (Fig. 2C), but LysBT1 differs from T7 lysozyme in that it is highly stable and active at pH 5.0. Although the molecular basis for acid resistance of LysBT1 remains to be elucidated, the tolerance of the enzyme to citric acid and EDTA, two OM permeabilizers, allows the enzyme to be used as an antibacterial agent against Gram-negative bacteria.

### Thermostability

Similar to reported thermophilic (pro)phage-derived endolysins, LysBT1 is highly thermostable. We found that the thermostability of LysBT1 is influenced by multiple factors including salinity, buffer type, enzyme concentration, EDTA, reductant, and oxidant, providing important implications for the stabilization mechanism of the enzyme.

Solvent-accessible salt bridges and long-range ion pairs are well known to contribute to protein stability at high temperatures (52). LysBT1 possesses a large number of surface ion pairs, and the presence of NaCl decreases the thermostability of the enzyme, most likely due to the weakening of stabilizing electrostatic interactions with increased ionic strength. Additionally, LysBT1 is more thermostable in the Tris buffer than in the phosphate buffer, probably because the Tris buffer has a lower ionic strength than the phosphate buffer and thus facilitates the strengthening of the electrostatic interactions between ion pairs.

Given that both LysBT1 and ΔSLH could form oligomers, it is reasonable that higher enzyme concentrations will promote oligomerization and stabilization of the enzymes. The protein concentration-dependent thermostabilization has not been reported for other endolysins yet, although some of them are verified to be oligomeric (49, 53–57). It would be interesting to determine whether the thermostabilities of these oligomeric endolysins also depend on protein concentration, which may be helpful for regulating enzyme stability and activity in a dose-dependent manner during the application of endolysins.

Compared with LysBT1, the catalytic Zn^2+^-coordinating residue variant C156S shows a remarkable decrease in heat resistance at 95°C. Similarly, the Zn^2+^-coordinating residue variants of Ts2631 also display a decreased thermostability (21). Meanwhile, EDTA treatment could destabilize LysBT1, Ts2631 (21), and PhiKo (23) at elevated temperatures. These evidence suggest that the catalytic Zn^2+^ plays an important role in the thermostabilization of the three thermophilic (pro)phage-derived NALAA with an Amidase_2 domain. A relatively high concentration of EDTA (10 mM) is needed to destabilize LysBT1 at 85°C, supporting the presence of a high-affinity binding site for the catalytic Zn^2+^. By contrast, at a relatively low concentration (0.1 mM), EDTA does not destabilize LysBT1 but improves its thermostability. Considering that LysBT1 has a high content of potential metal ion-binding residues (e.g., 16 Asp, 18 Glu, 8 His, and 7 Cys in 261 residues), we postulate that in addition to binding the catalytic Zn^2+^, LysBT1 may nonspecifically bind certain metal ions capable of disturbing the stabilizing forces such as the surface ion pairs and the interaction between the catalytic Zn^2+^ and its coordinating residues. In this case, the removal of nonspecifically bound metal ions by low concentration of EDTA would reinforce the stabilizing interactions of LysBT1, while a further increase of EDTA concentration would destabilize the enzyme by chelating the high-affinity catalytic Zn^2+^ from its coordinating residues.

Oxidation of side chains of some amino acid residues (e.g., Cys, Met, and His) is known to cause thermoinactivation in proteins (43, 58). At high temperatures, LysBT1 could be destabilized by H_2_O_2_ but stabilized by β-Me and DTT, indicating that reducing agents can prevent thermally induced oxidation and improve the thermostability of the enzyme. Given that nonspecifically bound metal ions may destabilize LysBT1, it is also possible that thiol groups of β-Me and DTT could chelate the destabilizing metal ions to improve enzyme stability. Similar to LysBT1, the Amidase_2 family endolysin PlyG is also thermostabilized by reducing agents (59). Although the residues Cys65, Cys73, Cys109, Cys110, and Cys211 of LysBT1 are not involved in disulfide bond formation or catalytic Zn^2+^ coordination, the individual substitution of these Cys residues by Ser destabilizes the enzyme. The replacement of the thiol group of Cys by the hydrophilic hydroxyl group of Ser possibly affects the structural stability of the enzyme via altering thiol-mediated hydrogen bonds and hydrophobic interactions (60).

The high thermostability of LysBT1 makes the enzyme suitable for applications at high temperatures. For instance, thermophilic *G. stearothermophilus* is a predominant spoilage bacterium in the food and feed industry (25). LysBT1 shows a high lytic activity toward *G. stearothermophilus*, representing an attractive candidate for use in food and feed processing involving heat treatment.

### Roles of the SLH domain

The SLH domain represents a unique structural feature of LysBT1 compared to other endolysins. LysBT1 is more thermostable than ΔSLH, indicating that the SLH domain contributes to the thermostability of the enzyme. Given that the SLH domain could mediate the trimerization of LysBT1, it appears that the SLH domain stabilizes LysBT1 through promoting the oligomerization of the enzyme. It is noteworthy that, similar to LysBT1, ΔSLH could also form oligomers, and its thermostability is also protein concentration dependent. This raises the possibility that LysBT1 oligomer is more stable than ΔSLH oligomer, emphasizing the role of the SLH domain in stabilizing the enzyme. In addition, the flexible linker between the NALAA and SLH domains in LysBT1 may enable the two domains to interact with each other to stabilize the enzyme.

Besides the structural similarity, the SCWP-binding motifs identified in the SLH domains of Sap and SpaA (16, 17) are highly conserved in that of LysBT1, indicating that these SLH domain-containing proteins are anchored to SCWPs in a similar manner. We noticed that the binding ligand of the SLH domain of LysBT1 can be removed from the cell surface of Gram-positive *G. stearothermophilus* or Gram-negative *E. coli* BL21(DE3) by EDTA. However, EDTA is unable to release SCWP that is covalently linked to peptidoglycan (61) from bacterial cells but is generally used as a removal agent of S-layer or OM. Considering that the SLH domains of Sap and SpaA bind to SCWPs through the terminal monosaccharide residue (16, 17), we postulate that the S-layer glycan of *G. stearothermophilus* (61) and the OM lipopolysaccharide of *E. coli* BL21(DE3) could serve as the ligand for the SLH domain of LysBT1. Nevertheless, not all of the Gram-positive or Gram-negative bacteria tested here could be bound by SLH domain of LysBT1, likely reflecting species-dependent variation in the composition of SCWP, S-layer glycan, or lipopolysaccharide.

The contribution of the SLH domain to LysBT1 activity depends on reaction conditions and bacterial species. At low enzyme concentrations, the SLH domain-mediated binding of LysBT1 to the cell surface of strain WF146 could increase local enzyme concentration to enhance enzymatic hydrolysis of peptidoglycan, while this enhancement effect would be weakened with the increase of enzyme concentration in the reaction mixture or the extension of the reaction time. Meanwhile, there is no strict linkage between the presence of the SLH domain and the antibacterial spectrum of the enzyme. Because the SLH domain is not essential for lytic activity of LysBT1, it is not astonishing that the enzyme could lyse the bacteria without binding affinity for the SLH domain. In the case of *B. cereus*, although it could be bound by the SLH domain of LysBT1, in either the absence or presence of EDTA, this bacterium could be lysed by LysBT1 only in the presence of EDTA. Most likely, in the absence of EDTA, the S-layer glycan of *B. cereus* acts as the ligand for LysBT1, but the enzyme is unable to access the peptidoglycan due to the molecular sieving effect of the S-layer (11). The EDTA-induced disorganization of the S-layer allows LysBT1 to bind SCWP and facilitates the enzyme to reach and act on the peptidoglycan of *B. cereus*. Given the wide distribution of S-layer among bacteria, the combinative use of LysBT1 and EDTA is expected to expand the antibacterial spectrum of the endolysin.

## MATERIALS AND METHODS

### Bacterial strains and growth conditions

The following bacteria were used for lytic activity assay and SLH domain binding assay: *B. thermoruber* WF146 (CCTCC AB209297), *G. stearothermophilus* (CCTCC AB92070), *B. subtilis* DB104 (CCTCC AB209263), *B. cereus* (CCTCC AB2010134), *S. aureus* (CCTCC AB2019136), *P. aeruginosa* (CCTCC AB2018157), *A. baumannii* (CCTCC AB2018157), and *E. coli* BL21(DE3) (serotype O7:H^-^). *B. thermoruber* WF146 and *G. stearothermophilus* were cultured at 55°C in Luria-Bertani (LB) medium, while *A. baumannii* was cultured at 37°C in a nutrient broth (NB) medium containing 10 g peptone, 3 g beef extract, 5 g NaCl per liter (pH 7.2). All other strains were cultured at 37°C in LB medium. *E. coli* DH5α and *E. coli* BL21(DE3) were used as the hosts for cloning and expression, respectively, and kanamycin (30 µg/mL) was added to the culture medium as needed.

### Plasmid construction and mutagenesis

The primers used for PCR are listed in Table S4. The genes encoding LysBT1 and its variants (ΔSLH and rSLH) were amplified from the genomic DNA of strain WF146 and inserted into the *Nde* I-*Xho* I site of pET26b (Novagen) to construct the expression plasmids (pET26b-*LysBT1*, pET26b-*ΔSLH*, and pET26b-*SLH*) in *E. coli* BL21(DE3). Previously constructed pET26b-*GFP* (36) was used to express recombinant GFP. The DNA fragments encoding GFP and the SLH domain were subcloned into pET26b using Ready-To-Use Seamless Cloning Kit (Sangon Biotech, Shanghai, China) to construct the plasmid pET26b-*GFP-SLH* for the fusion protein GFP-SLH. The QuikChange site-directed mutagenesis method (62) was employed to introduce point mutations into target proteins. All recombinant plasmids were confirmed using DNA sequencing.

### Expression and purification

*E. coli* BL21(DE3) cells harboring recombinant plasmids were grown at 37°C until the OD_600_ value reached ~0.7. The expression of recombinant proteins was induced by 0.4 mM IPTG and continued cultivation at 30°C for 4 h. The harvested cells were suspended in 50 mM Tris-HCl (pH 8.0) and disrupted by sonication on ice. The cell lysate was centrifuged at 13,000 × *g* for 10 min to recover the soluble cellular fraction. The recombinant protein with a fused His-tag in the soluble cellular fraction was purified by affinity chromatography on a Ni^2+^-charged chelating Sepharose Fast Flow resin (GE Healthcare) column, and the elution fractions were dialyzed against the Tris buffer (50 mM Tris-HCl, pH 7.4) or the phosphate buffer (50 mM NaH_2_PO_4_-NaOH, pH 7.4) overnight at 4°C. Protein concentrations were determined using the Bradford method with bovine serum albumin as a standard. The protein sample was concentrated with a Micron TM-3 centrifugal filter (Millipore, Bedford, MA, USA) as needed.

### SDS-PAGE, native-PAGE, and immunoblot analysis

Protein samples were precipitated with 20% (wt/vol) trichloroacetic acid (TCA), washed with ice-cold acetone, and then subjected to SDS-PAGE analysis. For native PAGE, SDS was absent during the whole process, and protein samples were not treated with TCA. The anti-His-tag monoclonal antibody (1:10,000, Novagen) and the goat anti-mouse IgG secondary antibody (1:5,000, Abbkine, China) were used for immunoblot analysis as described previously (63).

### Formaldehyde cross-linking

A total of 20 µg/mL of protein and 0.1%–1.0% (vol/vol) formaldehyde were mixed in 50 mM sodium phosphate buffer (pH 7.4) and incubated for 5 min at room temperature, followed by SDS-PAGE analysis.

### Lytic activity assay

The lytic activity of the enzyme was determined via turbidity reduction assay as previously described (21, 23) with modifications. Briefly, the exponential phase cells of *B. thermoruber* WF146 grown in LB medium were collected by centrifugation (4,000 × *g*, 10 min), washed twice, and resuspended in buffer A (50 mM NaH_2_PO_4_-NaOH, and 0.15 M NaCl, pH 7.4) with an OD_600_ of 1.4 to 1.6 (1 cm path length). Unless otherwise indicated, the determination of lytic activity was carried out using a single time-point assay at 37°C for 1 h in 1 mL reaction mixture containing 500 µL of preincubated cell suspension and 500 µL of preincubated enzyme sample in buffer A, followed by measurement of OD_600_ (experimental OD_600_). In the control sample, 500 µL of preincubated buffer A instead of the enzyme sample was mixed with the cell suspension, and the OD_600_ value was measured after incubation at 37°C for 1 h (control OD_600_). The lytic activity was calculated as follows: (control OD_600_ − experimental OD_600_)/initial OD_600_. In some cases, the change in OD_600_ of the reaction mixture was recorded over time at 37°C in a thermostated spectrophotometer (SP752; Shanghai Spectrum Instruments Co. Ltd, China) to determine the extent of bacteriolysis.

### Peptidoglycan hydrolytic activity assay

Peptidoglycan was extracted from *B. thermoruber* WF146 according to previously described method (64). Briefly, the exponential phase cells of strain WF146 were collected by centrifugation (4,000 × *g*, 10 min) from 500 mL of culture, washed with 0.8% (wt/vol) NaCl, resuspended in 30 mL of hot 4% SDS, boiled for 30 min, and then kept at room temperature (~25°C) overnight. Thereafter, the suspension was boiled for 10 min, cooled to room temperature, and then centrifuged at 15,000 × *g* for 15 min to collect the pellet containing peptidoglycan. The pellet was then washed five times with sterile deionized water and then collected by centrifugation (15,000 × *g*, 15 min).

Unless otherwise indicated, the peptidoglycan hydrolytic activity of the enzyme was determined 37°C for 1 h in 600 µL reaction mixture containing 300 µL of preincubated peptidoglycan suspension (OD_600_ = 1.3) and 300 µL of preincubated enzyme sample in buffer A, followed by measurement of OD_600_ (experimental OD_600_). In the control sample, the 300 µL enzyme sample was replaced by the same volume of buffer A, and the OD_600_ value was recorded after 1 h incubation at 37°C (control OD_600_). One unit of peptidoglycan hydrolytic activity was defined as the amount of enzyme required to decrease the OD600 value (ΔOD_600_ = control OD_600_ − experimental OD_600_) by 0.01 unit/min under the conditions described above.

### SLH domain binding assay

The bacterial cells were recovered from 1 mL of exponential phase culture by centrifugation (4,000 × *g*, 10 min), washed twice, and resuspended in 1 mL of the Tris buffer (50 mM Tris-HCl, pH 7.4). The bacterial suspension was supplemented with recombinant GFP, GFP-SLH, or its derivatives (0.16 µM) and incubated at 37°C for 1 h in the presence or absence of 10 mM EDTA. Thereafter, the cells were collected by centrifugation (4,000 × *g*, 5 min), washed twice with Tris buffer, resuspended in 1 mL of the same buffer, and examined by phase-contrast and fluorescence microscopy.

### Antibacterial activity assay

The exponential phase cells of different Gram-positive or Gram-negative bacteria were recovered by centrifugation (4,000 × *g*, 10 min), washed twice, and resuspended in the Tris buffer (50 mM Tris-HCl, pH 7.4). The bacterial suspensions with a final OD_600_ of 0.85 were incubated at 37°C for 1 h in the presence of LysBT1, ΔSLH, HEWL (Solarbio, Beijing, China), or polymyxin (Aladdin, Shanghai, China; 0.33 µM or 0.33 mM). In some cases, different concentrations of EDTA or citric acid were included in the reaction mixture. Thereafter, aliquots were serially diluted (1:10) and spotted on LB or NB (*A. baumannii*) agar plates to calculate viable cell numbers after overnight incubation at 37°C or 55°C (*G. stearothermophilus*).

### Bioinformatic analyses

The prophages were predicted using PHASTER (65). Structural models of proteins were predicted by AlphaFold (66). ChimeraX software (67) was used for visualization of the predicted structure. The distance limits were chosen to be 4.0 Å and 8.0 Å to find salt bridges (68) and long-range ion pairs (52), respectively. The phylogenetic tree was constructed using MEGA11 (69).

## Data Availability

The complete genome sequence of *Brevibacillus thermoruber* WF146 has been deposited in GenBank under the accession number PRJNA319752. The GenBank accession number of LysBT1 is WP_065068396.

## SUPPLEMENTAL MATERIAL

Supplemental material aem.00867-25-s0001.pdf Tables S1 to S4; Fig. S1 to S12.

ASM does not own the copyrights to Supplemental Material that may be linked to, or accessed through, an article. The authors have granted ASM a non-exclusive, world-wide license to publish the Supplemental Material files. Please contact the corresponding author directly for reuse.
