# Supplementary material for: Enzymatic property and stabilization mechanism of LysBT1, a novel polyextremotolerant endolysin with a C-terminal S-layer homology domain
Source: Appl Environ Microbiol. 2025 Jun 13;91(7):e00867-25. doi: 10.1128/aem.00867-25 (PMC12285257; doi:10.1128/aem.00867-25)
Supplement: Supplemental material — Tables S1 to S4; Fig. S1 to S12. [file aem.00867-25-s0001.pdf]

Supplementary Material

Enzymatic property and stabilization mechanism of LysBT1, a novel polyextremotolerant endolysin with a C-terminal S-layer homology domain

Yu Li,[a] Ke Luo,[a] Chaofeng Jiang,[a] Yihao Zhang,[a] Yong Yang,[a] Yitong Yao,[a] Huai Li,[a] Fei Gan,[b,c] # Xiao-Feng Tang,[b,c] # Bing Tang[a,c] #

[a]State Key Laboratory of Virology, College of Life Sciences, Wuhan University, Wuhan 430072, China

[b]Hubei Key Laboratory of Cell Homeostasis, College of Life Sciences, Wuhan University, Wuhan, China

[c]Cooperative Innovation Center of Industrial Fermentation (Ministry of Education & Hubei Province), Wuhan, China

Running title: A highly thermostable endolysin

#Address correspondence to Bing Tang, tangb@whu.edu.cn, Xiao-Feng Tang, tangxf@whu.edu.cn, or feigan@whu.edu.cn

Keywords: endolysin, N-acetylmuramoyl-L-alanine amidase, S-layer homology domain, prophage, thermophile, antimicrobials

TABLE S1 Charged amino acid content in LysBT1 and other Amidase_2 family endolysins

Endolysin	GenBank No.	pI	Number of residues							Charged residues%
			Total	D	E	D+E	K	R	K+R	
LysBT1	WP_065068396	8.8	261	16	18	34	23	17	40	28.4
Ph2119	AHF20915	9.6	155	6	7	13	10	9	19	20.6
Ts2631	AIM47292	10.0	156	4	9	13	8	14	22	22.4
LysAP45	YP_009831980	10.5	288	13	13	26	28	14	42	23.6
PhiKo	AYJ74695	9.7	171	6	9	15	9	12	21	21.1
T7 lysozyme	P00806	8.9	151	9	9	18	11	10	21	25.8
BlyA	AAC38300	10.1	367	19	10	29	39	11	50	21.5
XlyA	P39800	5.6	297	17	12	29	13	8	21	16.8
LysK	YP_009041293	9.9	495	20	18	38	45	16	61	20.0
PlyL	AAP27798	7.9	234	12	14	26	18	9	27	22.6
LysBPS13	YP_006907567	9.0	277	11	18	29	14	19	33	22.4
PlyPl23	AGN89335	6.1	224	13	15	28	12	10	22	22.3
LysVpKK5	AIM40565	6.8	163	11	10	21	8	10	18	23.9
PaAmi1	YP_009214894	8.5	285	18	10	28	16	17	33	21.4
LysCPS2	YP_009838139	8.8	226	15	9	24	18	10	28	23.0
ORF9	BAF81277	7.4	289	12	15	27	15	12	27	18.7
TM4 LysA	AAD17596	5.7	547	38	27	65	17	34	51	21.2
Ami1	WHT50385	9.3	334	17	19	36	33	13	46	24.6
LysB	ACD52487	7.5	224	12	14	26	20	6	26	23.2
CP25L	AGH27916	6.3	377	21	29	50	24	21	45	25.2
2638A	YP_239818	8.5	486	30	27	57	45	16	61	24.3
LysWMY	BAD834028	8.7	477	25	21	46	36	17	53	20.8
PlyTW	CAA69021	9.5	467	25	15	40	42	15	57	20.8
LysH5	B8QIR1	8.8	481	22	22	44	31	19	50	19.5
LysGH15	ADG26756	9.9	495	21	17	38	45	16	61	20.0
LysPBC2	AKQ08512	8.8	311	16	12	28	16	16	32	19.3
LysPW2	AZU98917	9.0	307	15	11	26	17	14	31	18.6
PlyG	ABB55421	8.5	233	13	12	25	20	8	28	22.7
Lc-Lys	YP_009282364	5.6	350	21	13	34	12	13	25	16.9
EJL	CAE82155	4.6	316	29	22	51	22	11	33	26.6
LysZ5	AEP68521	10.3	334	13	6	19	36	6	42	18.3
Ply511	YP_001468459	10.3	341	14	6	20	38	6	44	18.8

TABLE S2 Predicted ion pairs in LysBT1 monomer and those at the interfaces between SLH domains in LysBT1 trimer*

	LysBT1 monomer		Interfaces between SLH domains in LysBT1 trimer	
	Ion pair	Distance (Å)	Ion pair	Distance (Å)
Salt bridge	R4(NH2)-D67(OD1)	3.896	a/K221(NZ)-c/D248(OD1)	3.093
	R17(NH2)-E78(OE1)	3.369	a/D236(OD2)-c/R240(NH1)	3.424
	K23(NZ)-D102(OD2)	3.31	a/E242(OE1)-c/R240(NH2)	2.746
	R44(NH2)-E48(OE2)	3.759	a/R240(NH1)-b/D236(OD2)	3.501
	E48(OE1)-K51(NZ)	3.201	a/R240(NH2)-b/E242(OE1)	2.719
	E76(OE1)-R136(NH1)	2.955	a/D248(OD1)-b/K221(NZ)	3.090
	R95(NH2)-D143(OD1)	3.274	b/R240(NH2)-c/E242(OE1)	2.752
	E121(OE1)-R124(NH2)	3.841	b/R240(NH1)-c/D236(OD2)	3.444
	R169(NH2)-E173(OE1)	3.253	b/D248(OD1)-c/K221(NZ)	3.043
	K199(NZ)-E220(OE1)	3.892		
	E257(OE1)-R260(NH2)	3.486		
Long-range ion pair	R17(NH1)-E48(OE2)	7.172	a/K221(NZ)-c/D252(OD1)	4.120
	K26(NZ)-D102(OD1)	7.462	a/D252(OD1)-b/K221(NZ)	4.257
	K51(NZ)-D52(OD2)	6.009	b/D252(OD1)-c/K221(NZ)	4.105
	D52(OD2)-K54(NZ)	6.139		
	R95(NH1)-E149(OE1)	7.877		
	R98(NH2)-D102(OD2)	7.208		
	E108(OE1)-K154(NZ)	6.432		
	D113(OD2)-R117(NH1)	4.346		
	D115(OD2)-R117(NH2)	4.453		
	D115(OD1)-R158(NH2)	5.186		
	R124(NH1)-D188(OD2)	7.771		
	K128(NZ)-E177(OE1)	5.395		
	K135(NZ)-E177(OE1)	6.392		
	E141(OE1)-K179(NZ)	6.652		
	K148(NZ)-D155(OD1)	7.102		
	E149(OE1)-K171(NZ)	4.775		
	D155(OD2)-R158(NH2)	4.99		
	D166(OD1)-R169(NH1)	4.787		
	D166(OD1)-R172(NH2)	7.824		
	R169(NH2)-E187(OE2)	6.484		
	R169(NH2)-D188(OD1)	6.236		
	R172(NH2)-E173(OE1)	4.985		
	R172(NH2)-E176(OE1)	5.891		
	R172(NH2)-E187(OE2)	6.424		
	E176(OE1)-K184(NZ)	7.998		
	E176(OE1)-K179(NZ)	7.478		
	K184(NZ)-E187(OE1)	6.802		
	E187(OE2)-K189(NZ)	6.836		

	D188(OD2)-K190(NZ)	6.426
	K199(NZ)-D216(OD2)	7.436
	D203(OD2)-K233(NZ)	6.997
	K206(NZ)-E207(OE1)	7.847
	K206(NZ)-E209(OE2)	6.428
	E209(OE1)-K212(NZ)	7.594
	K212(NZ)-D213(OD1)	5.72
	K212(NZ)-D216(OD2)	4.38
	E220(OE2)-K221(NZ)	7.069
	D227(OD2)-K233(NZ)	4.317
	K233(NZ)-E242(OE1)	7.261
	K233(NZ)-D236(OD2)	4.745
	D248(OD2)-R249(NH2)	7.652
	D252(OD2)-K256(NZ)	6.267
	E257(OE1)-K261(NZ)	5.101

* The ion pairs involved in ionic networks are underlined. The three SLH domains in LysBT1 trimer are indicated by "a", "b", and "c", respectively.

TABLE S3 Antibacterial activity of LysBT1 and ΔSLH against *P. aeruginosa*

Additive	Log CFU/mL [a]		
	Control	LysBT1 (0.33 μM)	ΔSLH (0.33 μM)
-	8.54 ± 0.05	8.53 ± 0.18 (n.s.)	8.61 ± 0.11 (n.s.)
0.01 mM EDTA	8.29 ± 0.03	8.30 ± 0.01 (n.s.)	8.33 ± 0.11 (n.s.)
0.05 mM EDTA	7.20 ± 0.13	7.24 ± 0.11 (n.s.)	7.25 ± 0.04 (n.s.)
0.1 mM EDTA	4.91 ± 0.18	5.56 ± 0.48 (n.s.)	5.21 ± 0.10 (n.s.)
2 mM citric acid	8.24 ± 0.13	8.26 ± 0.11 (n.s.)	8.16 ± 0.01 (n.s.)
5 mM citric acid	3.57 ± 0.32	3.57 ± 0.09 (n.s.)	3.58 ± 0.17 (n.s.)

[a] Bacterial cells were incubated at 37°C for 1 h in the absence (control) or presence of LysBT1 or ΔSLH (0.33 μM) in the Tris buffer (50 mM Tris-HCl, pH 7.4) without (-) or with different concentration of EDTA or citric acid, and then spotted at 1:10 serial dilution on solid LB media for cell viability assay. The data are expressed as means ± SDs of three independent experiments. Statistically significant differences between the control and experimental samples were calculated by Student's *t* test (n.s., no significance).

TABLE S4 Primers used in this study

Primer	Sequence (5' to 3')*	Recombinant proteins
LysBT1-F	ATACATATGGAACTACGAGTCCAATCTAA	LysBT1, ΔSLH
LysBT1-R	AATCTCGAGTCA*ATGATGATGATGATGATG*TTTCCGCGTCAACTCCTTCA	LysBT1, rSLH, GFP-SLH
ΔSLH-R	AATCTCGAGTCA*ATGATGATGATGATGATG*GTATTTACTCATCAGATAAA	ΔSLH
SLH-F	ATACATATGCCCCCGGAGGACAAAAAAGC	rSLH
GFP-SLH-F	CTTTAAGAAGGAGATATACATATGAGTAAAGGAGAAGAA	GFP-SLH
GFP-SLH-GR-F	CTATACAAA**GGTAGCGGCGGTGGCGGTAGC**CCC	GFP-SLH
GFP-SLH-GR-R	**GCCGCTACC**CTTGTATAGTTCATCCATGCCATG	GFP-SLH
GFP-SLH-S-F	**GGTAGCGGCGGTGGCGGTAGC**CCCCCGGAGGACAAAAAAGC	GFP-SLH
H31A-F	GTCATCGCATGGGTCGCGAACCCAGGCAGTACCGCGCTC	H31A
H31A-R	GACCCATGCGATGACGACACCTTTAACGGGTTTCAAACG	H31A
E108A-F	GGAATCGCATGCTGCCATACGGATTGGGACGGGCGGATG	E108A
E108A-R	GCAGCATGCGATTCCGATAGTGCAATCGTTAGGGTACCG	E108A
H147A-F	TGGCTTGCAAAGGAAGTCGTCGGCTGGAAGGATTGTCAC	H147A
H147A-R	TTCCTTTGCAAGCCACAGGTCATTCTCGGTCAACCCGTA	H147A
K154A-F	GGCTGGGCAGATTGTCACCGCTGGTTCGTGAACAACCCG	K154A
K154A-R	ACAATCTGCCCAGCCGACGACTTCCTTGTGAAGCCACAG	K154A
C156A-F	AAGGATGCACACCGCTGGTTCGTGAACAACCCGGCTGAC	C156A
C156A-R	GCGGTGTGCATCCTTCCAGCCGACGACTTCCTTGTGAAG	C156A
C65S-F	ATCGTGAGCCTCGACGGGTCCATCGTTCAATGCCTGCCG	C65S, C65/109/110S
C65S-R	GTCGAGGCTCACGATATAGTGCGCGGACCCGTAACCGGA	C65S, C65/109/110S
C73S-F	GTTCAAAGCCTGCCGGAAACGGAAATGGCGTACCACGTC	C73S
C73S-R	CGGCAGGCTTTGAACGATGGACCCGTCGAGGCACACGAT	C73S
C103S-F	AACGATAGCACTATCGGAATCGAATGCTGCCATACGGAT	C103S
C103S-R	GATAGTGCTATCGTTAGGGTACCGGGATAGCCGCGTAAG	C103S
C109S-F	ATCGAAAGCTGCCATACGGATTGGGACGGGCGGATGA	C109S
C109S-R	ATGGCAGCTTTCGATTCCGATAGTGCAATCGTTAGGGTA	C109S
C110S-F	GAATGCAGCCATACGGATTGGGACGGGCGGATGA	C110S
C110S-R	CGTATGGCTGCATTCGATTCCGATAGTGCAATCGTTAGG	C110S
C156S-F	AAGGATAGCCACCGCTGGTTCGTGAACAACCCGGCTGAC	C156S
C156S-R	GCGGTGGCTATCCTTCCAGCCGACGACTTCCTTGTGAAG	C156S
C211S-F	GAGTGGAGCAAGGATCACGCGGACTCTCTGTACGAGAAG	C211S
C211S-R	ATCCTTGCTCCACTCCAGCTCTTTGGGAATGTCCTTGAA	C211S
C109/110S-F	ATCGAAAGCAGCCATACGGATTGGGACGGGCGGATGA	C109/110S, C65/109/110S
C109/110S-R	CGTATGGCTGCTTTCGATTCCGATAGTGCAATCGTTA	C109/110S, C65/109/110S
R240A-F	ACCGACCCGTTATCGGCTGCGGAGGCCGCCGTG	GFP-S-R240A
R240A-R	CACGGCGGCCTCCGCAGCCGATAACGGGTCGGT	GFP-S-R240A
E242A-F	CCGTTATCGCGCGCGGCTGCCGCCGTGTTGATC	GFP-S-E242A
E242A-R	GATCAACACGGCGGCAGCCGCGCGCGATAACGG	GFP-S-E242A

* Underlined sequences indicate restriction enzyme sites. The linker-coding sequences in GFP fusion proteins are in boldface. The italicized section indicates the His6 tag-coding DNA sequence. The mutated nucleotides are boxed.

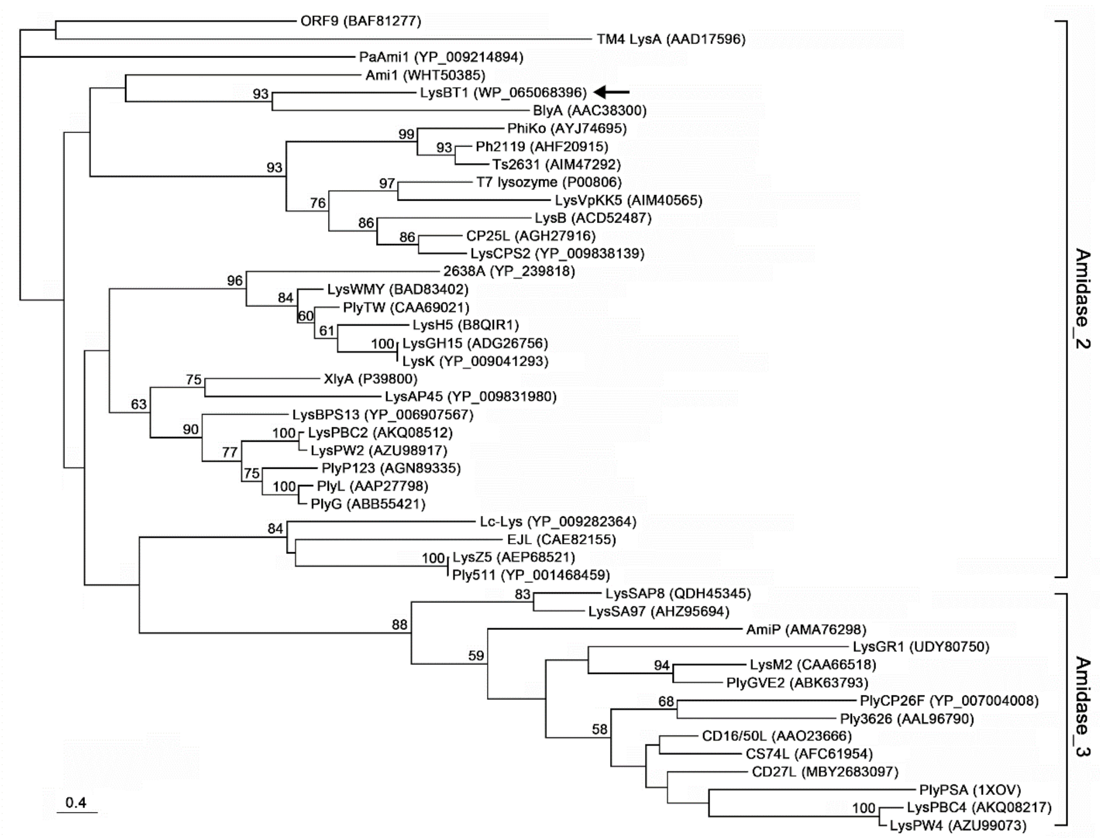

FIG S1 Phylogenetic analysis of endolysins belonging to Amidase_2 and Amidase_3 families. The GenBank accession numbers are listed in parentheses. LysBT1 is indicated by an arrow. Numbers at nodes indicate bootstrap values (percentages of 1,000 replicates), and only the values above 50% are shown.

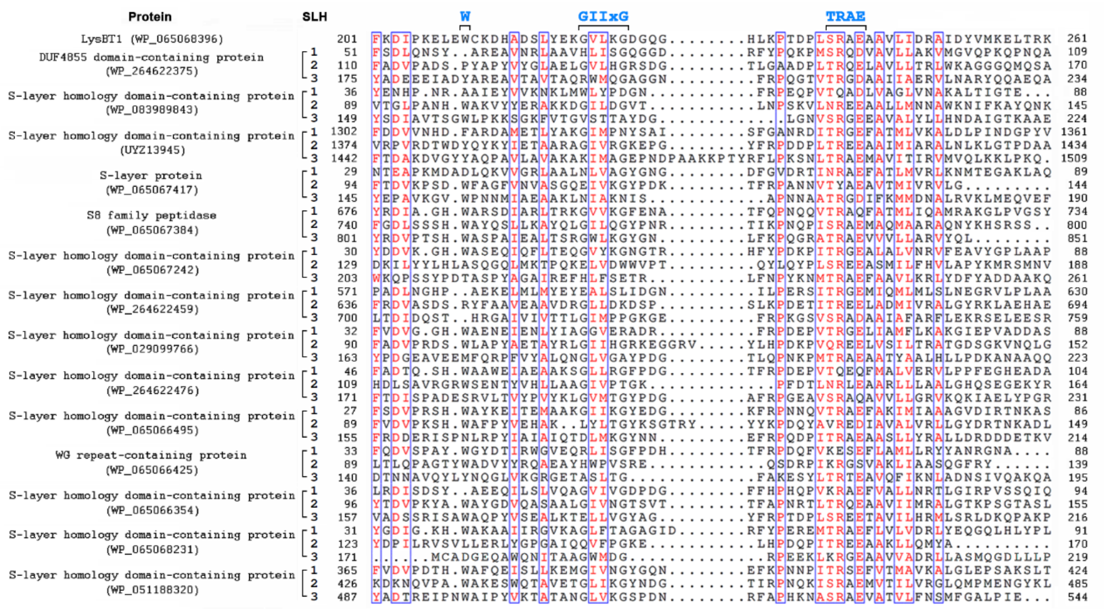

FIG S2 Alignment of SLH domains of LysBT1 and the additional 14 SLH domain-containing proteins in strain WF146. GenBank accession numbers of the SLH domain-containing proteins are shown in parentheses. The three tandem SLH domains in each protein are indicated by “1”, “2”, and “3”, respectively. The conserved Trp residue (W), the GIIxG motif, and the TRAE motif involved in binding SCWPs are marked.

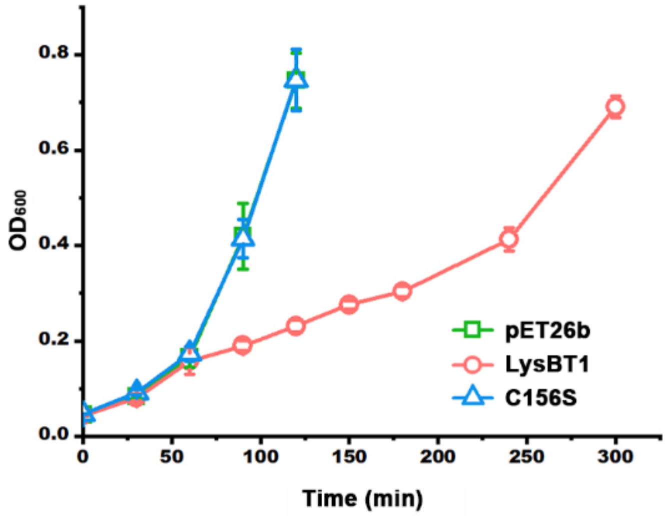

FIG S3 Growth curves of *E. coli* strains carrying pET26b or expression plasmid for target protein. The strains were grown at 37°C in LB media containing 30 µg/ml of kanamycin. The data are expressed as means ± standard deviations (SDs) of three independent experiments.

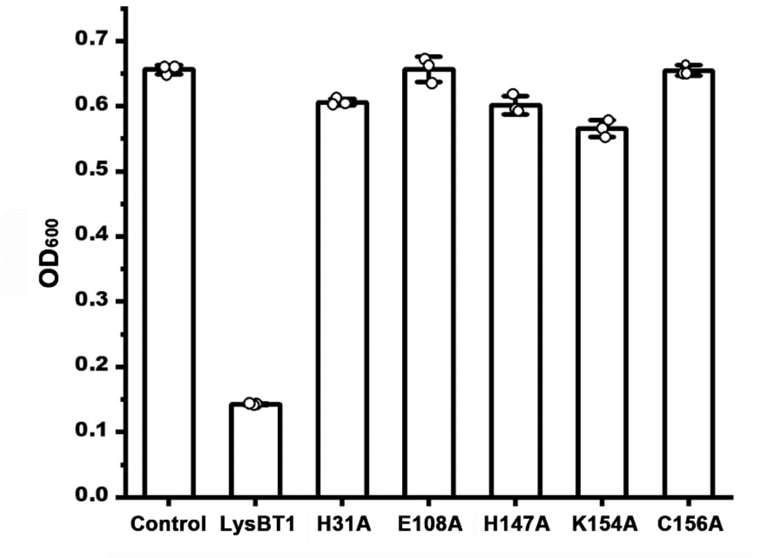

FIG S4 Lysis of strain WF146 cells by LysBT1 and its active-site variants. Strain WF146 cells were incubated at 37°C in the phosphate buffer (50 mM NaH_2PO_4-NaOH, pH 7.4) without (Control) or with the enzyme (0.33 μM) for 1 h, followed by OD_{600} measurement. The data are expressed as means ± standard deviations (SDs) of three independent experiments.

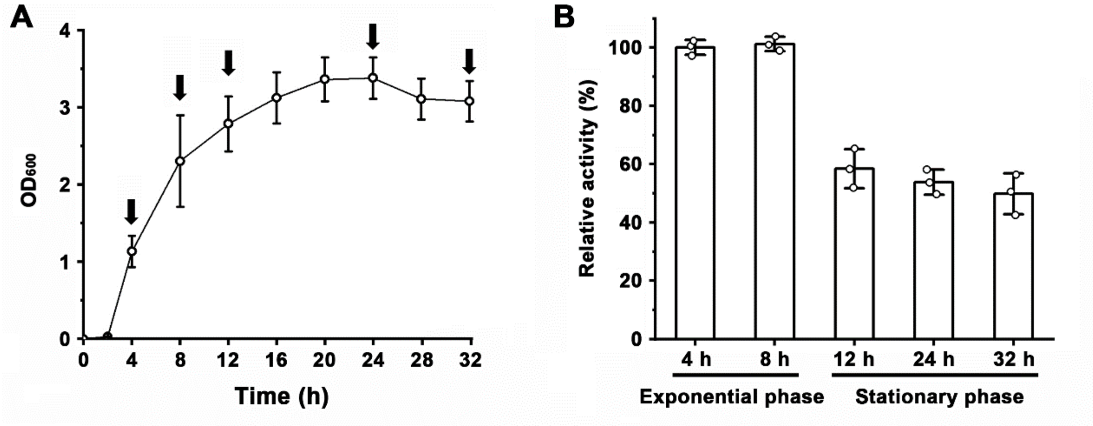

FIG S5 Lytic activity of LysBT1 against strain WF146 cells at different growth phases. (A) The growth curve of strain WF146 cultured at 55°C in LB medium. The arrows indicate the sampling time points. (B) Lytic activity of the enzyme. Strain WF146 cells at different growth phases were used as the substrates to determine the lytic activity of LysBT1 (0.33 μM) at 37°C for 1 h in the phosphate buffer (50 mM NaH_2PO_4-NaOH, pH 7.4) containing 0.15 M NaCl. Relative activity was calculated by defining the highest activity among the data set as 100%. The data are expressed as means ± standard deviations (SDs) of three independent experiments.

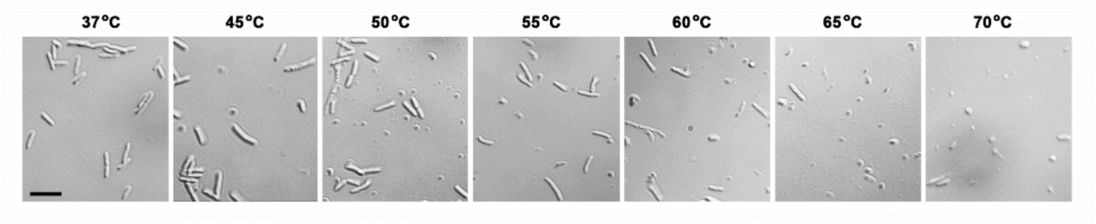

FIG S6 Effect of temperature on morphology of *B. thermoruber* WF146 cells. Strain WF146 cells were incubated at different temperatures for 5 min in the phosphate buffer (50 mM NaH_2PO_4-NaOH, pH 7.4) containing 0.15 M NaCl, followed by phase-contrast microscopy. Bar, 5 μm.

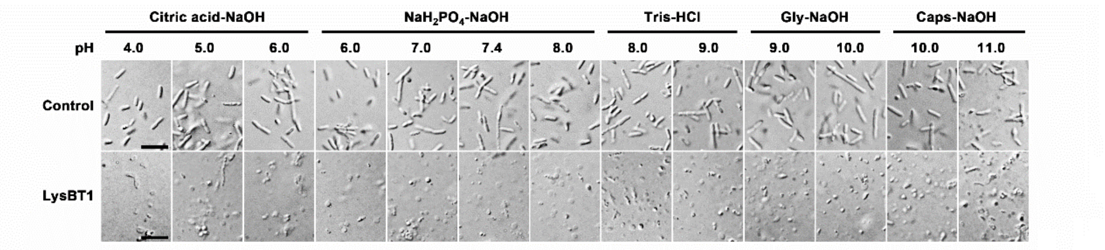

FIG S7 Effects of pH values on LysBT1-mediated lysis of *B. thermoruber* WF146 cells. Strain WF146 cells were incubated alone (Control) or with LysBT1 (0.13 μM) at 37°C for 1 h in 50 mM of different buffers (containing 0.15 M NaCl) with different pH values, followed by phase-contrast microscopy. Bars, 5 μm.

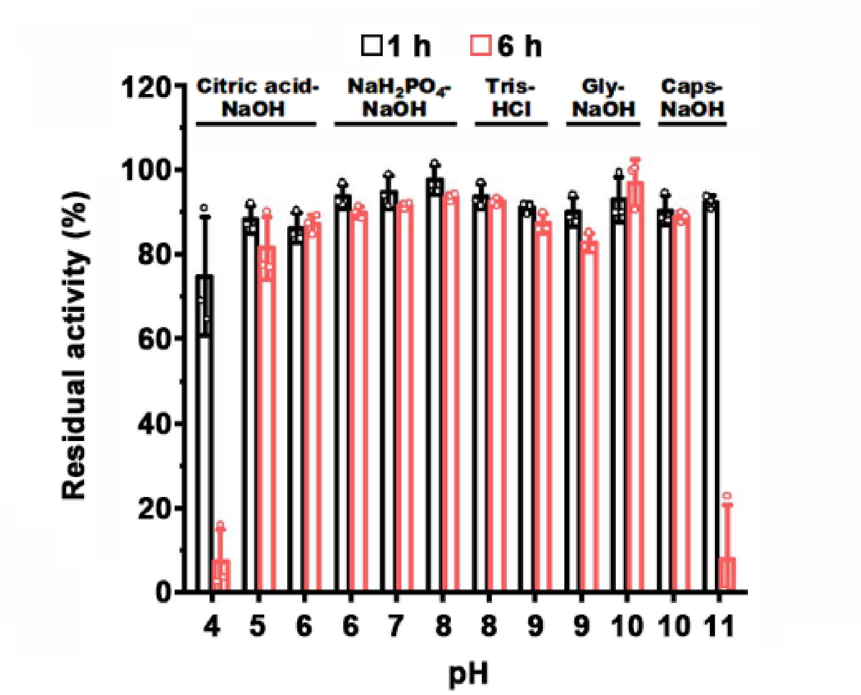

FIG S8 pH stability of the enzyme. The enzyme (0.13 μM) was incubated at 37°C in the indicated buffers (50 mM) containing 0.15 M NaCl. At the indicated time periods, samples were taken and diluted 1:10 in the phosphate buffer containing 0.15 M NaCl for lytic activity assay performed at 55°C for 5 min.

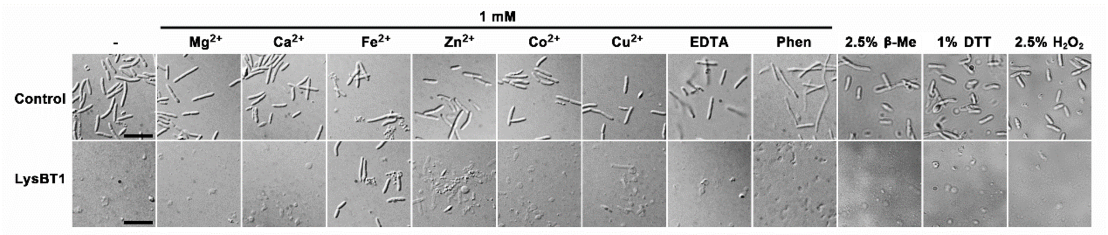

FIG S9 Effects of metal ions, chelators, and redox agents on LysBT1-mediated lysis of *B. thermoruber* WF146 cells. Strain WF146 cells were incubated alone (Control) or with LysBT1 (0.33 μM) at 37°C for 1 h in the Tris buffer (50 mM Tris-HCl, pH 7.4) containing the indicated agent, followed by phase-contrast microscopy. Bars, 5 μm.

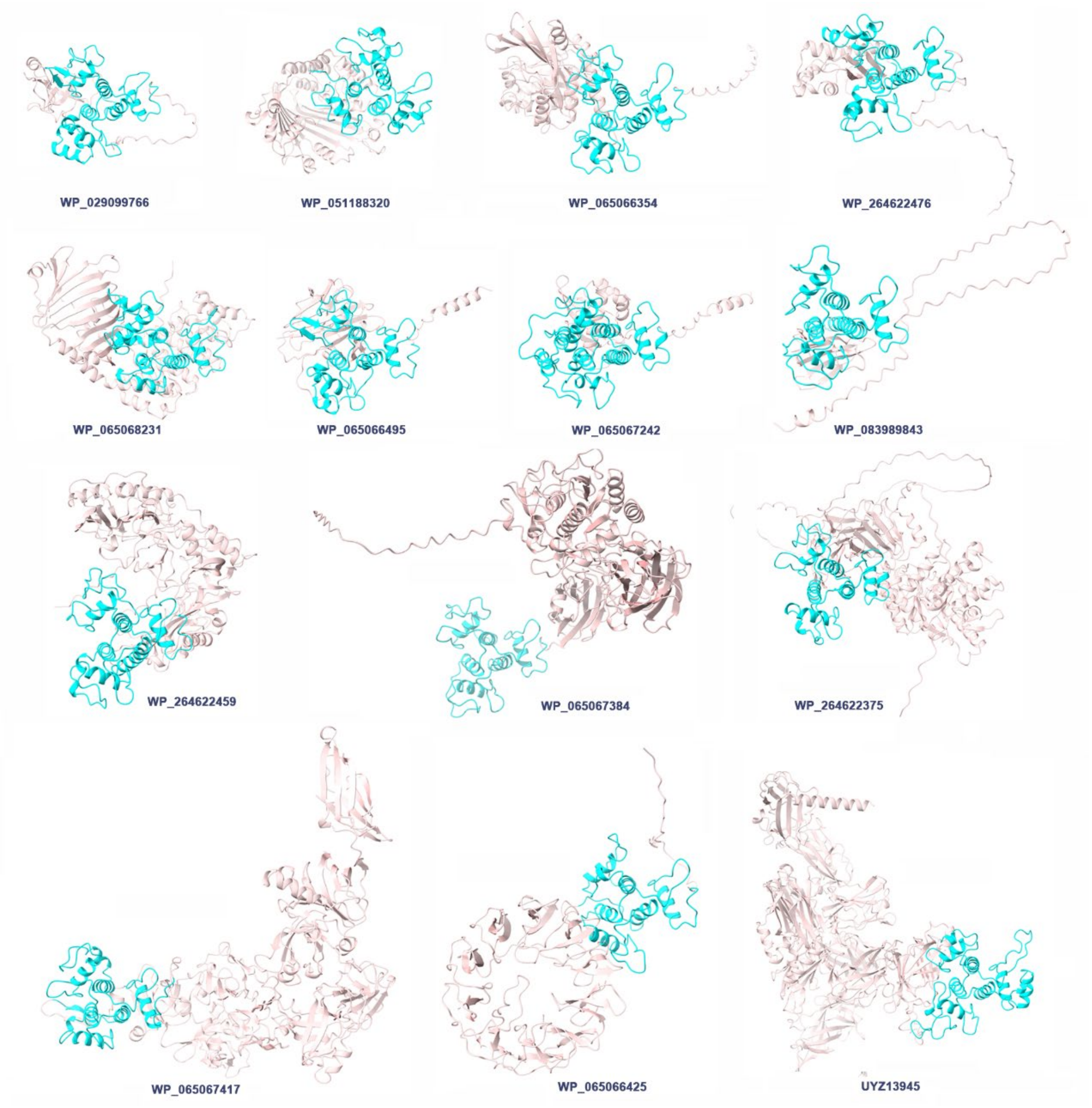

FIG S10 Predicted structures of the additional 14 SLH domain-containing proteins of strain WF146. The pseudotrimer composed of three tandem SLH domains in each protein is indicated in blue.

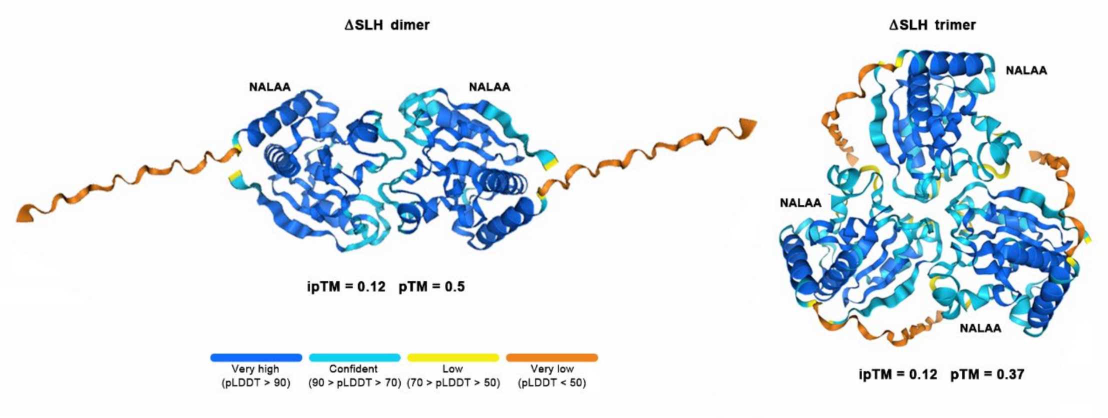

FIG S11 Predicted structures of the dimer and trimer of ΔSLH.

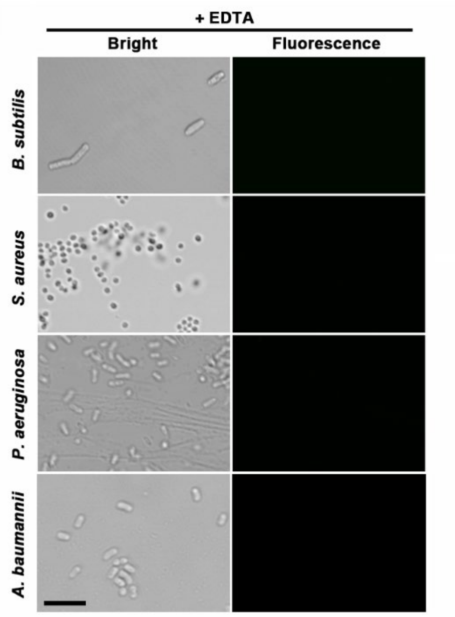

FIG S12 Cell surface-binding capacity of the SLH domain of LysBT1. Bacterial cells were incubated with recombinant GFP-SLH (0.16 μM) at 37°C for 1 h in the Tris buffer (50 mM Tris-HCl, pH 7.4) with (+) 10 mM EDTA, washed with the same buffer, and examined by phase-contrast and fluorescence microscopy. Bar, 5 μm.
